# Hierarchical deconvolution for extensive cell type resolution in the human brain using DNA methylation

**DOI:** 10.3389/fnins.2023.1198243

**Published:** 2023-06-19

**Authors:** Ze Zhang, John K. Wiencke, Karl T. Kelsey, Devin C. Koestler, Annette M. Molinaro, Steven C. Pike, Prasoona Karra, Brock C. Christensen, Lucas A. Salas

**Affiliations:** ^1^Department of Epidemiology, Geisel School of Medicine, Dartmouth College, Lebanon, NH, United States; ^2^Department of Neurological Surgery, Institute for Human Genetics, University of California, San Francisco, San Francisco, CA, United States; ^3^Department of Epidemiology, Department of Pathology and Laboratory Medicine, Brown University School of Public Health, Providence, RI, United States; ^4^Department of Biostatistics and Data Science, University of Kansas Medical Center, Kansas City, KS, United States; ^5^Department of Neurology, Geisel School of Medicine, Dartmouth College, Lebanon, NH, United States; ^6^Department of Molecular and Systems Biology, Geisel School of Medicine, Dartmouth College, Lebanon, NH, United States

**Keywords:** DNA methylation, deconvolution, epigenetics, brain heterogeneity, brain deconvolution

## Abstract

**Introduction:**

The human brain comprises heterogeneous cell types whose composition can be altered with physiological and pathological conditions. New approaches to discern the diversity and distribution of brain cells associated with neurological conditions would significantly advance the study of brain-related pathophysiology and neuroscience. Unlike single-nuclei approaches, DNA methylation-based deconvolution does not require special sample handling or processing, is cost-effective, and easily scales to large study designs. Existing DNA methylation-based methods for brain cell deconvolution are limited in the number of cell types deconvolved

**Methods:**

Using DNA methylation profiles of the top cell-type-specific differentially methylated CpGs, we employed a hierarchical modeling approach to deconvolve GABAergic neurons, glutamatergic neurons, astrocytes, microglial cells, oligodendrocytes, endothelial cells, and stromal cells.

**Results:**

We demonstrate the utility of our method by applying it to data on normal tissues from various brain regions and in aging and diseased tissues, including Alzheimer’s disease, autism, Huntington’s disease, epilepsy, and schizophrenia.

**Discussion:**

We expect that the ability to determine the cellular composition in the brain using only DNA from bulk samples will accelerate understanding brain cell type composition and cell-type-specific epigenetic states in normal and diseased brain tissues.

## Introduction

The human brain is arguably the most complex organ regarding its cellular composition and diversity (Guillaumet-Adkins and Heyn, 2017). Understanding the cellular heterogeneity and complexity of the brain is fundamental; indeed, assessment of brain cell alteration in neurological and psychiatric disorders plays a critical role in underlying the disturbance of brain cellular homeostasis. For instance, neuronal cell loss is one hallmark of Alzheimer’s disease (Serrano-Pozo et al., 2011). Recent studies using single-cell RNA sequencing (scRNA-seq) technology have revealed the landscape of brain cell diversity (Guillaumet-Adkins and Heyn, 2017; Mu et al., 2019). Genome and transcriptome profiling in individual brain cells has enabled the disentangling of its complex cellular composition. However, due to the heterogeneity of brain cells within different regions, the results from scRNA-seq vary widely by the regions where sc-RNA seq technology has been applied (Guillaumet-Adkins and Heyn, 2017; Mu et al., 2019). Although scRNA-seq provides insights and promising findings that are beginning to define this brain cell heterogeneity, the precise cell composition landscape of the brain remains incomplete. Flow cytometry technologies like fluorescence-activated cell sorting (FACS) have been used to sort a heterogeneous mixture of cells, e.g., immune cells (Leavitt et al., 2017; An and Chen, 2018; Milward et al., 2019). However, challenges involving cell components’ isolation, especially for the complex composition of cell populations like the brain, impede the use of FACS to understand its cell heterogeneity (Guez-Barber et al., 2012; Crouch and Doetsch, 2018). Other challenges in making accurate sorting of brain cells complex include destroying cells during sorting and postmortem autolysis, as human brain samples are typically acquired postmortem (Suarez-Pinilla and Fernandez-Vega, 2015; Cossarizza et al., 2017). Some studies have overcome sorting challenges by identifying nuclei markers as proxies for cell identification (Marcilla et al., 2001; Tsuchiya et al., 2003; Shi et al., 2004). Researchers have used neuronal nuclei (NeuN) as a biomarker for identifying neuronal cells (Gusel'nikova and Korzhevskiy, 2015). Although this approach can distinguish neuronal brain cells from non-neuronal brain cells, individual brain cell heterogeneity is not captured. A more direct, DNA-based approach to accurately deconvolute brain cell-type composition from bulk tissues can overcome challenges in studying brain cell type heterogeneity in brain-related disorders, including neurodegenerative diseases and cancer.

DNA methylation is an epigenetic modification that regulates gene expression and is essential to establish and preserving cellular identity (Bogdanovic and Lister, 2017). Genome-wide DNA methylation arrays provide a standardized and cost-effective approach to measuring DNA methylation. When combined with a cell-type reference library, DNA methylation measures allow the assessment of underlying cell-type proportions in heterogeneous mixtures (Salas et al., 2018, 2022). In recent years, DNA methylation has been widely utilized as a biomarker of immune cell types to infer cellular composition (Titus et al., 2017; Salas et al., 2022). Initially, using differentially methylated regions identified between purified leukocyte subtypes, we developed a reference-based deconvolution algorithm to estimate the distribution of subtypes of leukocytes in whole blood samples (Houseman et al., 2012). We later optimized the library by developing the IDOL algorithm and expanding the immune cell types in the library (Koestler et al., 2016; Salas et al., 2018, 2022). Methods now enable referenced-based libraries for estimating cell composition in the tumor microenvironment (Zhang et al., 2022), skin (Muse et al., 2022), and biospecimens from the breast (Muse et al., 2023).

Like in other human tissues, the identity of brain cell types is preserved in epigenomic markers, including DNA methylation (Kozlenkov et al., 2018; Rizzardi et al., 2019). Previous research devised two major approaches for brain cell deconvolution. Guintivano et al. developed a reference-based algorithm, cell epigenotype specific (CETS) marks, to quantify neuronal and non-neuronal cell proportions in brain samples utilizing the differential DNA methylation identified between neuronal and non-neuronal cells (Guintivano et al., 2013). Teschendorff et al. developed the EpiSCORE algorithm, which uses single-cell RNA-seq constructed DNA methylation libraries for multiple tissue deconvolution, including brain (Teschendorff et al., 2020; Zhu et al., 2022). EpiSCORE achieved deconvolution for six brain cell types. Here, we introduce a reference-based method using a hierarchical modeling approach with differential DNA methylation patterns among seven major brain cell types, Hierarchical Brain Extended Deconvolution (HiBED), for estimating cell-type proportions in brain samples. The reference libraries are based upon DNA methylation identities preserved in GABAergic (inhibitory) neurons (GABA), glutamatergic (excitatory) neurons (GLU), astrocytes, microglial cells, oligodendrocytes, endothelial cells, and stromal cells. We demonstrate that application of HiBED uncovers brain cell heterogeneity in various regions and alterations of brain cell distribution in aging and brain-related disorders.

## Materials and methods

All analyses were performed using R version 4.2.0.

### Discovery data sets

We used five publicly available data sets containing DNA methylation data on purified brain cells to construct our brain deconvolution libraries (Table 1). The discovery data sets included isolated samples from human primary astrocytes from the post-mortem sub-ventricular deep white matter (Weightman Potter et al., 2021; *n* = 6), endothelial cells from the cord tissue (Lin et al., 2018; *n* = 12), GABAergic neurons (GABA; Kozlenkov et al., 2018; *n* = 5) and glutamatergic neurons (GLU; Kozlenkov et al., 2018; *n* = 5) from the post-mortem dorsolateral prefrontal cortex, microglial cells from the post-mortem medial frontal gyrus, superior temporal gyrus, subventricular zone and thalamus (de Witte et al., 2022; *n* = 18), oligodendrocytes from the post-mortem Brodmann area 46 (Mendizabal et al., 2019; *n* = 20), and stromal cells from the cord tissue (Lin et al., 2018; *n* = 14). Due to the lack of age and sex information for astrocytes, Horvath methylation age and inferred sex were calculated using *ENMIX* (Xu et al., 2016) and *SeSAMe* (Zhou et al., 2018), respectively. DNA methylation data on oligodendrocytes was generated from whole-genome bisulfite sequencing (WGBS), while the rest was from either Illumina methylation 450 K or EPIC bead array. To integrate the data from different platforms, we used *methyLiftover* (Titus et al., 2016), which maps DNA methylation data from bisulfite sequencing to CpG sites measured with Illumina methylation bead-array platforms on oligodendrocyte WGBS data. After integrating the data, we performed beta-mixture quantile normalization (BMIQ) to normalize the methylation value using ChAMP (Teschendorff et al., 2013; Tian et al., 2017). We removed cross-reactive probes, SNP-related probes, sex chromosome probes, and non-CpG probes from the analysis. The final data set for identifying cell-type-specific DNA methylation included 80 samples. After integration, normalization, and removal of missing values, the data set consisted of 309,287 CpGs.

**Table 1:** Baseline characteristics of the discovery data sets.

Cell type	*N*	Mean age (sd)	*n* Male (%)	Accession	Source	Platform
Astrocyte	6	27.4 (11.2)*	6 (100)^#^	GSE166845 (29)	GEO	EPIC
Endothelial	12	Newborn	8 (66.7)	FlowSorted.CordTissueAndBlood.EPIC (30)	R package	EPIC
GABA	5	24.6 (3.7)	5 (100)	syn4588488 (24)	Synpase	450 K
GLU	5	24.6 (3.7)	5 (100)	syn4588488	Synpase	450 K
Microglia	18	83 (17.8)	4 (22.2)	GSE191200 (31)	GEO	EPIC
Oligodendrocyte	20	55.7 (15.8)	13 (65)	GSE107729 (32)	GEO	WGBS
Stromal	14	Newborn	10 (71.4)	FlowSorted.CordTissueAndBlood.EPIC	R package	EPIC
Total	80					

* Horvath methylation age was inferred using the ENMIX due to the lack of age information.

# Sex was inferred using the SeSAMe package due to the lack of sex information.

### HiBED hierarchy and brain cell-type-specific CpG identification

**Figure 1:**
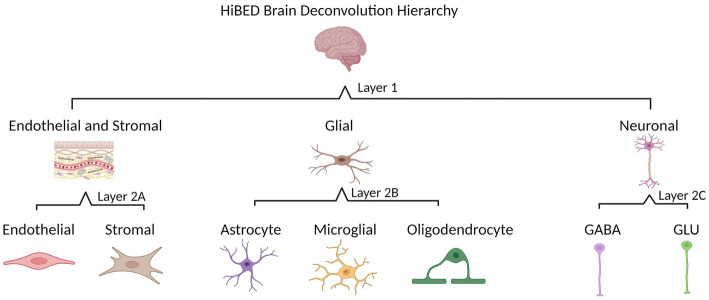
HiBED brain deconvolution hierarchy with four layers for seven brain cell types (Created with BioRender.com).

The HiBED deconvolution hierarchy was established based on cellular identification in the human brain. Two layers with four categories were set for seven brain cell types in the hierarchy (Figure 1). Layer 1 contains three cell groups (glial, neuronal, and endothelial and stromal cells). Layer 2A includes endothelial and stromal cells. Layer 2B has three glial cell types (astrocyte, microglia, and oligodendrocyte). Layer 2C includes two neuronal cell types (GABA and GLU). We used an adaptation of the *meffil.cell.type.specific.methylation* function in the *perishky/meffil* package (Min et al., 2018), which used linear regression with empirical Bayes adjustment statistics to reduce methylation profiles to most cell-type-specific sites to identify discernible CpGs in each layer and category across brain cell subpopulations within the category. One library is generated for each category in the hierarchy.

We tested nine sets of libraries with multiple methylation directions and various numbers of CpGs per cell type generated by parameter specification in the function. Libraries with hypermethylated CpG loci only, hypomethylated CpG loci only, and hyper and hypomethylated CpG loci hybrid were initially created. For each direction, 50, 100, and 200 CpG loci ranked by t-statistics were included in the libraries. We compared the dispersion separability criterion (DSC) among seven brain cell sub-populations across libraries to evaluate the performance among the nine libraries (Bell-Glenn et al., 2022). The results suggested the best overall performance of the library with 50 hybrid CpG loci per cell type (Supplementary Figure S1). We also used the absolute difference between actual and estimated values using the libraries to assess the performance. The library with 50 hybrid CpG loci per cell type consistently demonstrated the best performance with the lowest absolute difference value (Supplementary Figure S2). Thus, the libraries with 50 hybrid CpG loci per cell type were selected as the reference libraries for HiBED deconvolution. Heatmaps illustrate the differential methylation state between cell types across the four layers. InfiniumMethylation BeadChips Annotation file was used to map the CpGs in the libraries to the associated genes (Zhou et al., 2017). UCSC Genome Browser was used to investigate the CpG location relative to the association gene (Kent et al., 2002). The libraries were then used in conjunction with the constrained projection quadratic programming approach described by Houseman et al. to estimate the proportions of brain cell types (Houseman et al., 2012). There are three steps established in HiBED to project brain cell proportions. First, the Layer 1 library was used to estimate the proportions of neurons, glial cells, and endothelial and stromal cells. Second, Layer 2A, 2B, and 2C libraries were used to estimate endothelial cell and stromal cell, neuronal cell types (GABA and GLU), and glial cell types (astrocyte, oligodendrocyte, microglia) respectively. Third, the cell proportions estimated from Layer 1 libraries were deconvoluted to respective cell proportions by weighting the cell proportions estimated from Layer 2 libraries. The HiBED deconvolution function was then created in the HiBED package in R with 2 layers of deconvolution that is user-specifiable. The first layer outputs estimated cell proportions for neuronal, glial, and endothelial and stromal cells. The second layer outputs estimated cell proportions for GABA, GLU, astrocyte, oligodendrocyte, microglia, endothelial cell, and stromal cell. HiBED can be applied to WGBS and methylation microarray data. For WGBS data, the users need to use *methyLiftover* to convert the WGBS data to microarray data first (Titus et al., 2016). The HiBED package is available at.^1^

### HiBED validation

We generated 50 *in silico* synthetic mixtures of brain cell subpopulations to validate the libraries using 80 purified brain cell samples from the discovery data sets (Table 1). Seven random numbers were generated from a uniform distribution and the proportions for those numbers were calculated and assigned to seven cell types for each sample. The 50 artificial samples contain a gradient of cell proportions for each cell type. The cell-type-specific DNA methylation matrices were generated by randomly sampling the purified brain cell samples for each cell type and averaging the methylation beta value for each probe. Standard deviations were calculated from randomly selected purified samples. The artificial bulk brain sample DNA methylation matrices were then generated by multiplying predefined cell proportion matrices and purified methylation matrices. The projected proportion was investigated for correlation with the expected proportion for each cell type. Pearson’s correlation and root mean squared error were used to evaluate the performance. For external validation, we applied the algorithm to a previously used neuronal cell projection DNA methylation data set on GEO (GSE41826) that included 58 sorted neuronal and 58 non-neuronal nuclei samples from post-mortem frontal cortex, 9 *in silico* neuronal mixture with a gradient neuron proportion 10% increase, and 20 bulk brain samples with fluorescence-activated nuclei sorting (FANS) measured neuronal proportion (Guintivano et al., 2013; Table 2). We also tested HiBED on microglial samples from bipolar, schizophrenia, and major depression disorder patients with an age range 21–93 (de Witte et al., 2022) and adult vein endothelial cells (Sarkar et al., 2020; Table 2). The performance of HiBED was compared with CETS and EpiSCORE on the *in silico* neuronal mixture, the FANS-measuered bulk brain samples, and the diseased microglia samples.

**Table 2:** Baseline characteristics of the external validation data sets.

Sample	*N*	Mean age (sd)	*n* Male(%)	GEO accession
NeuN+	58	32.05 (15.84)	28 (48.3)	GSE41826 (26)
NeuN-	58	32.05 (15.84)	28 (48.3)	
Mix	9	50.00 (0.00)	9 (100.0)	
Bulk	20	37.20 (19.37)	10 (50.0)	
Microglia	36	60 (22.7)	16 (44.4)	GSE191200 (31)
Vein endothelial cell	8	48.3 (0.9)	0 (0)	GSE142439 (42)
Total	145			

### HiBED application

We identified 516 samples from 11 publicly available GEO data sets containing DNA methylation data on the normal brain with various sub-regions (Table 3; Pidsley et al., 2013, 2014; De Souza et al., 2016; Horvath et al., 2016; Watson et al., 2016; Murphy et al., 2017; Viana et al., 2017; Gasparoni et al., 2018; Smith et al., 2018). To make a more general comparison across the brain, we collapsed those regions into four significant subgroups, cerebellum, basal ganglia, hippocampus, and cortex. The Infinium signal intensity files were pooled across the data sets, and beta-mixture quantile normalization (BMIQ) was employed for data processing. With HiBED, we proceeded to deconvolve brain cells in the application data sets and demonstrate the brain cell composition with 7 HiBED cell types across the regions using notched boxplots and stacked bar plots. We also calculated the glia-to-neuron ratio (GNR) and showed the distribution of GNR across the brain regions. Glial cell composition with HiBED glial cells (astrocyte, microglia, oligodendrocyte) was demonstrated in the cortex region. Furthermore, we interrogated the aging effect on cell proportions within brain sub-regions by correlating the predicted cell proportions with age in the cerebellum, basal ganglia, hippocampus, and cortex. Pearson’s correlation coefficient was used to demonstrate the relationship between age and cell proportions.

**Table 3:** Baseline characteristics of the application data sets with normal brain samples from various regions.

Sample	*N*	Mean age (sd)	*n* Male (%)	GEO accession
Cerebellum	40	63.15 (20.04)	26 (65.0)	GSE43414 (43), GSE61431 (47), GSE72778 (44)
Hippocampus	20	63.55 (18.27)	14 (70.0)	GSE72778, GSE89703 (46)
**Basal ganglia**				
caudate nucleus	12	56.33 (17.52)	10 (83.3)	GSE72778
midBrain	2	45.50 (10.61)	1 (50.0)	GSE72778
**Cortical structure**				
frontal cortex	180	66.14 (21.40)	113 (62.8)	GSE43414, GSE61380 (47), GSE61431, GSE66351 (48), GSE72778, GSE80970 (51), GSE88890 (50)
temporal pole	154	77.10 (14.35)	80 (51.9)	GSE43414, GSE66351, GSE67419 (50), GSE72778, GSE76105 (49), GSE80970
cingulate cortex	30	47.27 (19.96)	24 (80.0)	GSE72778, GSE88890
entorhinal cortex	5	76.00 (10.61)	3 (60.0)	GSE43414
motor cortex	12	56.33 (17.52)	10 (83.3)	GSE72778
visual cortex	23	55.26 (16.67)	19 (82.6)	GSE72778
inferior parietal	11	54.09 (16.46)	9 (81.8)	GSE72778
superior parietal	12	56.33 (17.52)	10 (83.3)	GSE72778
occipital pole	9	62.89 (25.79)	5 (55.6)	GSE72778
cortex	6	62.17 (21.83)	6 (100)	GSE79064 (45)
Total	516			

Next, we applied the deconvolution algorithm to four independent GEO data sets containing DNA methylation data on neurological and psychiatric disorders and control samples (Table 4). The disease data sets included 16 multiple Alzheimer’s disease samples and 14 controls in basal ganglia, 22 autism samples and 23 controls in the cortex, 197 Huntington’s disease and control samples in basal ganglia and cortex, 19 epilepsy samples and 14 controls in cortex and hippocampus, and 20 schizophrenia samples and 23 controls in cortex (Nardone et al., 2014; Pidsley et al., 2014; Horvath et al., 2016; Martins-Ferreira et al., 2022). For consistency, we performed BMIQ normalization on methylation beta values for those data sets. Four hundred and sixty-seven samples were eventually contained in the disease datasets. We compared HiBED cell proportion differences by brain region between diseased and control samples. Multivariable linear regression models were used to adjust for sex and age. Due to the lack of age information for epilepsy and Autism data sets, Horvath methylation age was calculated using *ENMIX* (Xu et al., 2016).

**Table 4:** Baseline characteristics of the application data sets with diseased brain samples and their corresponding normal controls.

Condition	*N*	Mean age (sd)	*n* Male (%)	Brain region	GEO accession
Alzheimer’s disease	16	93.31 (19.58)	4 (25.0)	Basal ganglia	GSE72778 (44)
Healthy control	14	54.79 (16.84)	11 (78.6)	Basal ganglia	GSE72778
Autism	22	36.27 (7.05)*	22 (100.0)	Cortex	GSE53924 (52)
Healthy control	23	38.01 (10.82)*	23 (100.0)	Cortex	GSE53924
Huntington’s disease	197	56.14 (14.85)	124 (62.9)	Basal ganglia, Cortex	GSE72778
Healthy control	119	56.13 (18.16)	90 (75.6)	Basal ganglia, Cortex	GSE72778
Epilepsy	19	39.43 (7.67)*	10 (52.6)	Cortex, Hippocampus	GSE168916 (53)
Healthy control	14	63.04 (10.76)*	10 (71.4)	Cortex, Hippocampus	GSE168916
Schizophrenia	20	62.05 (15.87)	11 (55.0)	Cortex	GSE61431 (47)
Healthy control	23	62.04 (18.74)	17 (73.9)	Cortex	GSE61431
Total	467				

* Horvath methylation age was inferred using the ENMIX due to the lack of age information.

## Results

### HiBED development

We used multiple validated genome-wide DNA methylation data sets on purified brain cell populations. Five publicly available data sets that contain DNA methylation data from 80 GABA, GLU, astrocytes, microglial cells, oligodendrocytes, endothelial cells, and stromal cells were used as the discovery data sets (Table 1). After integrating the data with quality control, we performed genome-wide differential methylation analyses on 309,287 CpGs to identify our deconvolution libraries.

Four libraries were developed based on the brain cell hierarchical tree (Figure 1). In Layer 1, 81 CpGs were identified to discern three major brain cell groups. In Layer 2A, 183 CpGs were specified to distinguish endothelial and stromal cells. In Layer 2B, 237 CpGs were identified to discern glial cell types. In Layer 2C, 120 CpGs were identified to distinguish neuronal cell types. The heatmaps in Supplementary Figure S3 demonstrated discriminative methylation status for the brain cell type-specific CpGs in the libraries. The libraries are relatively different, with 26 overlapping CpGs across the four libraries, 22 CpG appeared in 3 out of 4 libraries, and 18 CpGs in total overlapped in 2 out of 4 libraries (Supplementary Figure S4).

### Cell identity genes associated with HiBED

Among the genes associated with the CpGs in the libraries, we identified well-established genes related to cell identity. Cg08331427 is in an intron of *ECSCR* (Endothelial Cell Surface Expressed Chemotaxis And Apoptosis Regulator) and is a hypomethylated probe in the HiBED Layer 2A library distinguishing endothelial cells and other cell types (Supplementary Figure S5A)*. ECSCR* is expressed in endothelial cells and blood vessels, where it functions in cell shape changes and EGF-induced cell migration (Lu et al., 2012). Cg23165166 is in an intron of *COL5A2* (Collagen Type V Alpha 2 Chain) and marks hypomethylation in stromal cells compared to other cells in the HiBED Layer 2A library (Supplementary Figure S5B). *COL5A2* encodes an alpha chain for fibrillar collagens in stroma (Egusa et al., 2007). Cg19360930 is in an exon region of *WDR35* (WD Repeat Domain 35) and is hypomethylated in astrocyte compared to other cells in the HiBED Layer 2B library (Supplementary Figure S5C). *WDR35* has been found to have the highest expression in astrocytes across 80 cell types in RNA single-cell type specificity analysis from the Human Protein Atlas (Uhlen et al., 2015). Cg04341806 is in an intron of *LCP1* (Lymphocyte Cytosolic Protein 1) and hypomethylated in microglia compated to other cells in the HiBED Layer 2B library (Supplementary Figure S5D). *LCP1* is a myeloid marker for macrophage and microglial populations (Herbomel et al., 1999; Jin et al., 2019; Wu et al., 2020). Cg05578056 is in an intron of *LMF1* (Lipase Maturation Factor 1) and hypomethylated in oligodendrocytes compared to other cell types in the HiBED Layer 2B library (Supplementary Figure S5E). *LMF1* hallmarks the highest expression in oligodendrocyte precursor cells and oligodendrocytes across 80 cell types in RNA single-cell type specificity analysis from the Human Protein Atlas (Uhlen et al., 2015). Cg04812615 is in an intron of *PAK6* (P21 Activated Kinase 6) and hypermethylated in GABA compared to other cell types in the HiBED Layer 2C library (Supplementary Figure S5F). *PAK6* has been found to play a vital role in regulating morphological changes in GABAergic neuron development in the cortex (Goyette et al., 2019). Cg02632583 is located in an intron of *SLC38A10* (Solute Carrier Family 38 Member 10) and marks hypomethylation in GLU compared to other cells in the HiBED Layer 2C library (Supplementary Figure S5G). *SLC38A10* regulates glutamate homeostasis in cortex cells (Tripathi et al., 2022). The results showed that HiBED libraries captured critical functional genes that enabled discernibility between brain cell subpopulations.

### HiBED validation

**Figure 2:**
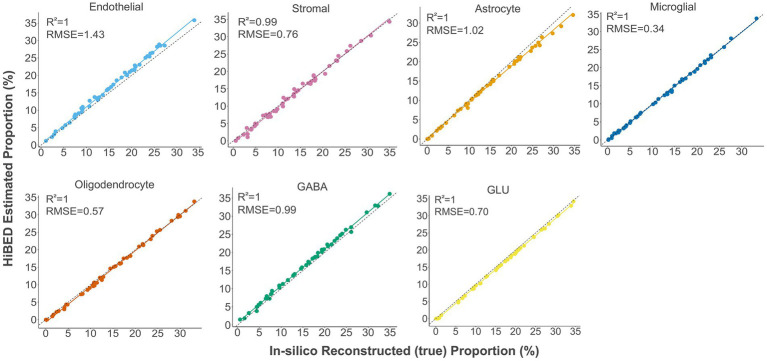
Internal validation of HiBED-predicted cell proportions with true reconstructed cell proportions in fifty *in silico* synthetic mixtures.

**Figure 3:**
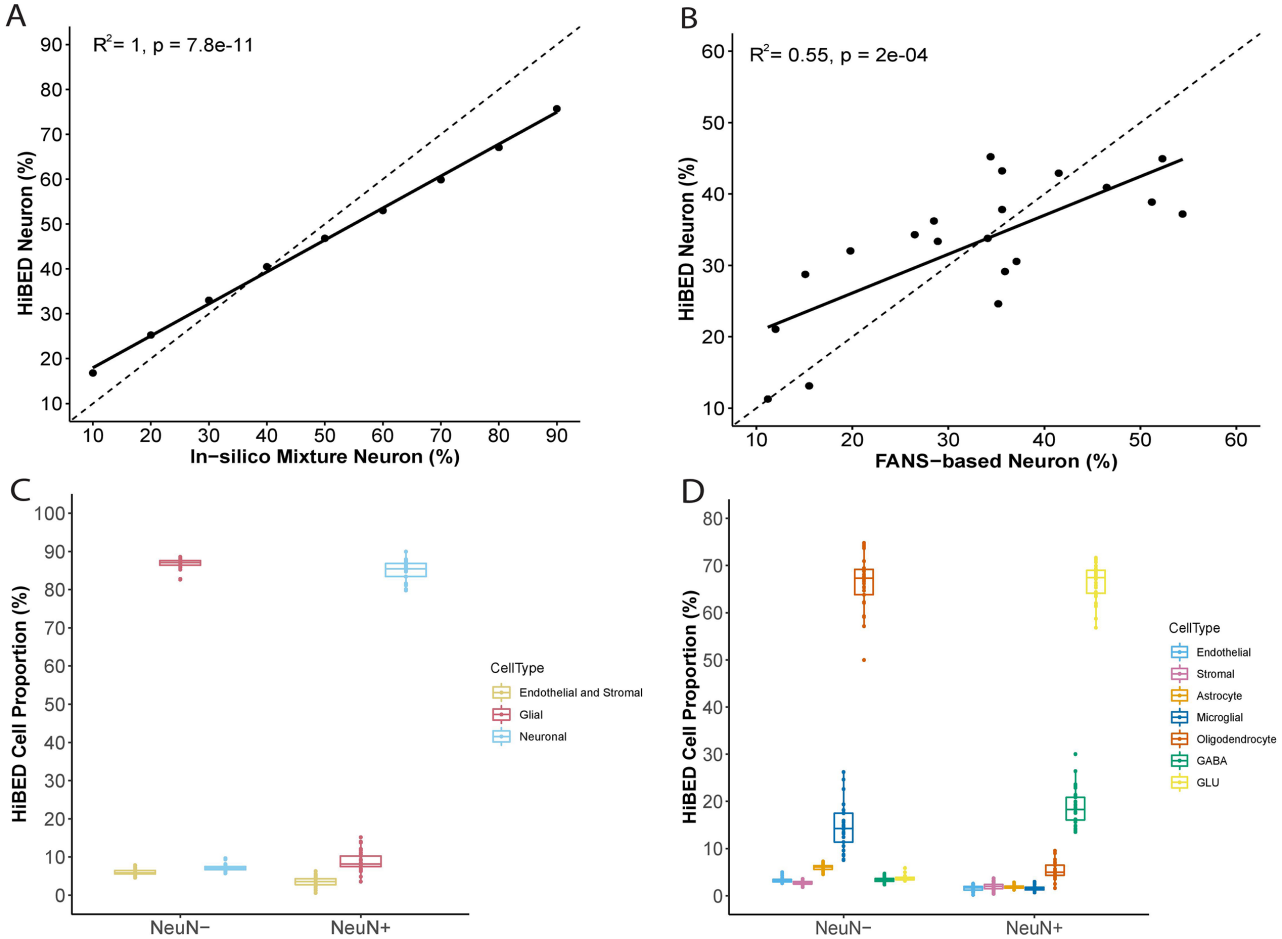
External validation of HiBED-predicted cell proportions. **(A)** Comparison of HiBED-predicted neuron proportions with in-silico mixture neuron proportions. **(B)** Comparison of HiBED-predicted neuron proportions with FANS-based neuron proportions in bulk brain samples. **(C)** HiBED Layer 1 deconvolution of sorted neuronal and non-neuronal nuclei samples. **(D)** HiBED Layer 2 deconvolution of sorted neuronal and non-neuronal nuclei samples.

The libraries were then used with the constrained projection/quadratic programming approach from Houseman et al. to estimate brain cell types’ proportions in mixed samples (Houseman et al., 2012). We first generated 50 *in silico* synthetic mixtures of brain cell subpopulations using the purified brain cell samples to validate the method. The predicted proportion was then tested for correlation with the expected proportion for each cell type. The results showed a high correlation and < 1% deviation between the predicted proportion and expected proportions for all cell types (average *R*^2^ = 1, average RMSE = 0.83%, Figure 2). As external validation, we used GSE41826, a publicly available data set (Table 2) with sorted neuronal and non-neuronal nuclei samples from postmortem frontal cortex, *in silico* neuronal mixture with a gradient increase in neuron proportion, and bulk brain samples with neuronal proportion data from fluorescence-activated nuclei sorting (FANS) on neuronal nuclei (Guintivano et al., 2013; Gusel'nikova and Korzhevskiy, 2015). The neuronal proportions were inferred by HiBED first-layer deconvolution. For the *in silico* neuronal mixture samples, we observed a high correlation between the HiBED estimated neuron proportion and expected neuron proportion (*R*^2^ = 1, *p* = 7.8e-11, Figure 3A). The HiBED performance on neuron projection is similar to previously described CETS neuron projection method (*R*^2^ = 1, *p* = 3.23e-10) and outperforms the EpiSCORE method (*R*^2^ = 0.95, *p* = 6.2e-06), although the CETS method consistently underestimates the true neuron proportion (Supplementary Figure S6A). For bulk brain samples, a significant positive correlation was noted between the estimated neuron proportion and FANS-measured neuron proportion (*R*^2^ = 0.55, *p* = 2e-04, Figure 3B), consistent with the CETS projection results (*R*^2^ = 0.61, *p* = 4.5e-05) and outperforms the EpiSCORE method (*R*^2^ = 0.61, *p* = 4.5e-05, Supplementary Figure S6B). HiBED Layer 1 deconvolution showed a predominant neuron proportion (Mean = 85.08%, SD = 2.46%) in sorted neuronal nuclei (NeuN+) samples and a substantial proportion of glial cells (mean = 86.81%, SD = 1.38%) in non-neuronal nuclei (NeuN-) samples (Figure 3C). HiBED Layer 2 deconvolution demonstrated higher proportions of GLU and GABA in NeuN+ samples (GLU: mean = 66.39%, SD = 4.80%; GABA: mean = 18.69%, SD = 3.87%) and relatively higher proportions of oligodendrocytes, microglia, and astrocytes compared to other cell types in NeuN- samples (oligodendrocyte: mean = 66.21%, SD = 5.59%; microglia: mean = 14.61%, SD = 4.74%; Astrocyte: 5.99%, SD = 0.61%, Figure 3D). HiBED demonstrated consistent high performance on microglial samples from patients with psychiatric disorders aging from 21 to 93 (Microglia: Mean = 97.04%, SD = 1.77%, Supplementary Figure S7A) and adult vein endothelial cells (Endothelial cell: Mean = 93.38%, SD = 5.14%, Supplementary Figure S7B). HiBED outperforms EpiSCORE on microglial samples from patients with psychiatric disorders (HiBED: microglia mean = 97.04%, SD = 1.77%; EpiSCORE: microglia mean = 49.98%, SD = 3.36%, Supplementary Figure S8). These results validated the HiBED libraries and showed that the projection algorithm based on the libraries could estimate the proportions of seven brain cell types in bulk brain samples.

### HiBED-profiled brain cell composition difference by region

**Figure 4:**
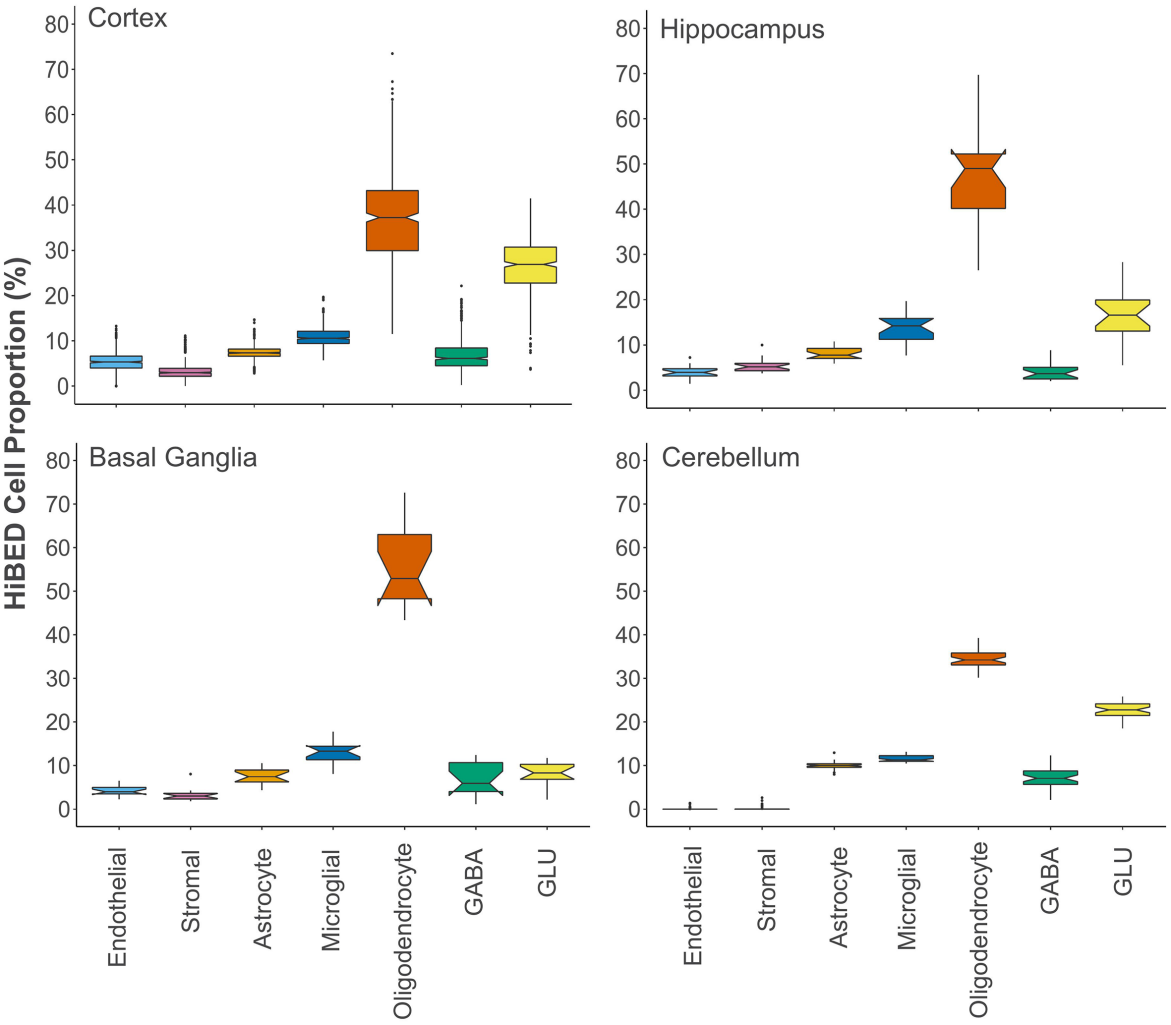
The distribution of HiBED-predicted brain cells in the cortex (*N* = 442), hippocampus (*N* = 20), basal ganglia (*N* = 14), and cerebellum (*N* = 40).

Next, we applied the algorithm to eleven publicly available independent data sets that contain DNA methylation data on regionally sampled normal brain tissues (Table 3). We interrogated 516 normal brain tissue samples from 14 different regions in the application data sets (Pidsley et al., 2013, 2014; De Souza et al., 2016; Horvath et al., 2016; Watson et al., 2016; Murphy et al., 2017; Viana et al., 2017; Gasparoni et al., 2018; Smith et al., 2018). To make a more general comparison across the brain, we collapsed those regions into four major groups, cerebellum, basal ganglia, hippocampus, and cortex. Notched boxplots showing the distribution of HiBED brain cells for the four regional groups were presented, respectively (Figure 4). Stacked bar plots were made to visualize the comparison of cell composition across brain sub-regions (Supplementary Figure S9). The results convey the variation and heterogeneity of the brain cell composition captured by HiBED across various regions.

### HiBED-profiled brain cell alteration with aging

**Figure 5:**
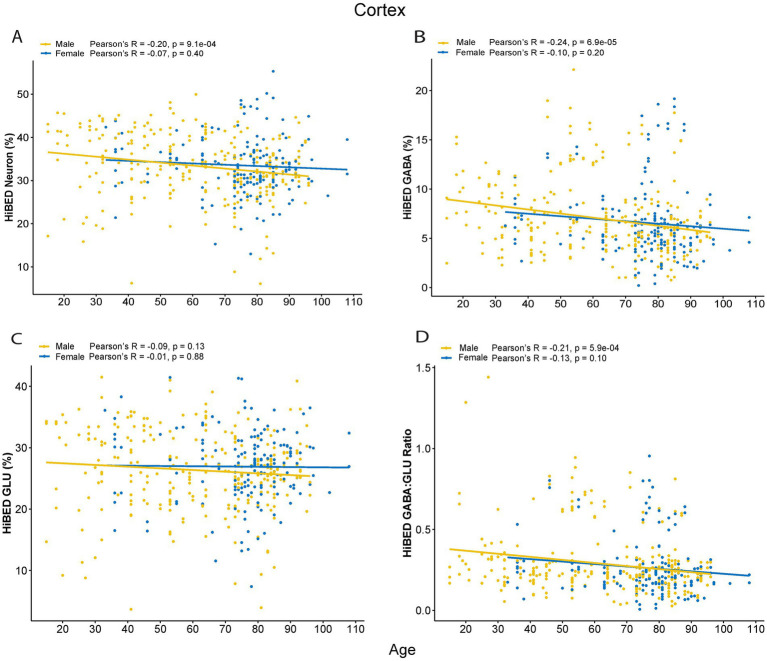
Aging effect on HiBED-predicted. **(A)** Neuron proportion. **(B)** GABA proportion. **(C)** GLU proportion and **(D)** GABA to GLU ratio in cortex stratified by sex (*N* = 442).

We then investigated the relationship of age with neuronal cell proportions per brain region. A weak but significant negative correlation was observed in the cortex between age and predicted neuron proportion (Pearson’s *r* = −0.15*, p* = 0.0018). However, when stratified by sex, only the male group showed a significant correlation (Pearson’s *r* = −0.20, *p* = 9.1e-04, Figure 5A). Among neuronal cell types, a stronger negative correlation between GABA and age was observed in the male group (Pearson’s *r* = −0.24, *p* = 6.9e-05, Figure 5B). In contrast, no significant association of age with GLU proportions was observed (Figure 5C). The GABA to GLU ratio also demonstrated a significant negative correlation with age in the male group (Pearson’s r = −0.21, *p* = 5.9e-04, Figure 5D). Considering the negative correlation between age and predicted GABA proportion in the male group in the cortex, when examined, the relationship stratified by cortical subregions. We observed that the negative aging effect on GABA was primarily driven by their strong negative correlation in the cingulate cortex (Pearson’s *r* = −0.61, *p* = 0.0016) and temporal pole (Pearson’s *r* = −0.51, *p* = 3.7e-06; Supplementary Figure S10). In the cerebellum, no significant correlations were observed between neuron proportions and age stratified by sex (Supplementary Figure S11A). However, a strong negative association of aging with GABA proportion (Pearson’s *r* = −0.41, *p* = 0.04, Supplementary Figure S11B) and a positive association of aging with GLU proportion (Pearson’s *r* = 0.51, *p* = 0.008, Supplementary Figure S11C) were observed in males. Also, a significant negative correlation between age and GABA to GLU ratio was observed in males (Pearson’s *r* = −0.45, *p* = 0.02, Supplementary Figure S11D). No significant correlations were observed between age and predicted neuron proportions in the hippocampus and basal ganglia, though these regions had smaller sample sizes.

### HiBED-profiled glia to neuron ratio

Quantification of the glia–neuron ratio (GNR) in the human central nervous system has been studied by multiple authors to understand the cellular composition, evolution of the brain, and brain-related pathophysiology (von Bartheld et al., 2016). Using the cell proportions projected from the algorithm, we explored the GNRs across the four major regions in the brain. We observed mean (sd) GNRs to be 6.97 (6.31), 1.89 (0.25), 1.86 (1.15), 3.74 (1.81), respectively, for basal ganglia, cerebellum, cortex, and hippocampus (Supplementary Figure S12). We also calculated the glial cell percentage composition in the cortex. We observed a mean (sd) 13.79% (4.15%), 20.17% (5.45%), and 66.04% (8.70%) contribution of astrocyte, microglia, and oligodendrocyte to glial cell constitution in the cortex (Supplementary Figure S13). The results suggest that the HiBED deconvolution method added a new layer to neuronal and glial cell estimation that enables more granular analyses of cellular constituency in the human brain.

### HiBED-profiled brain cell alteration with health conditions

**Figure 6:**
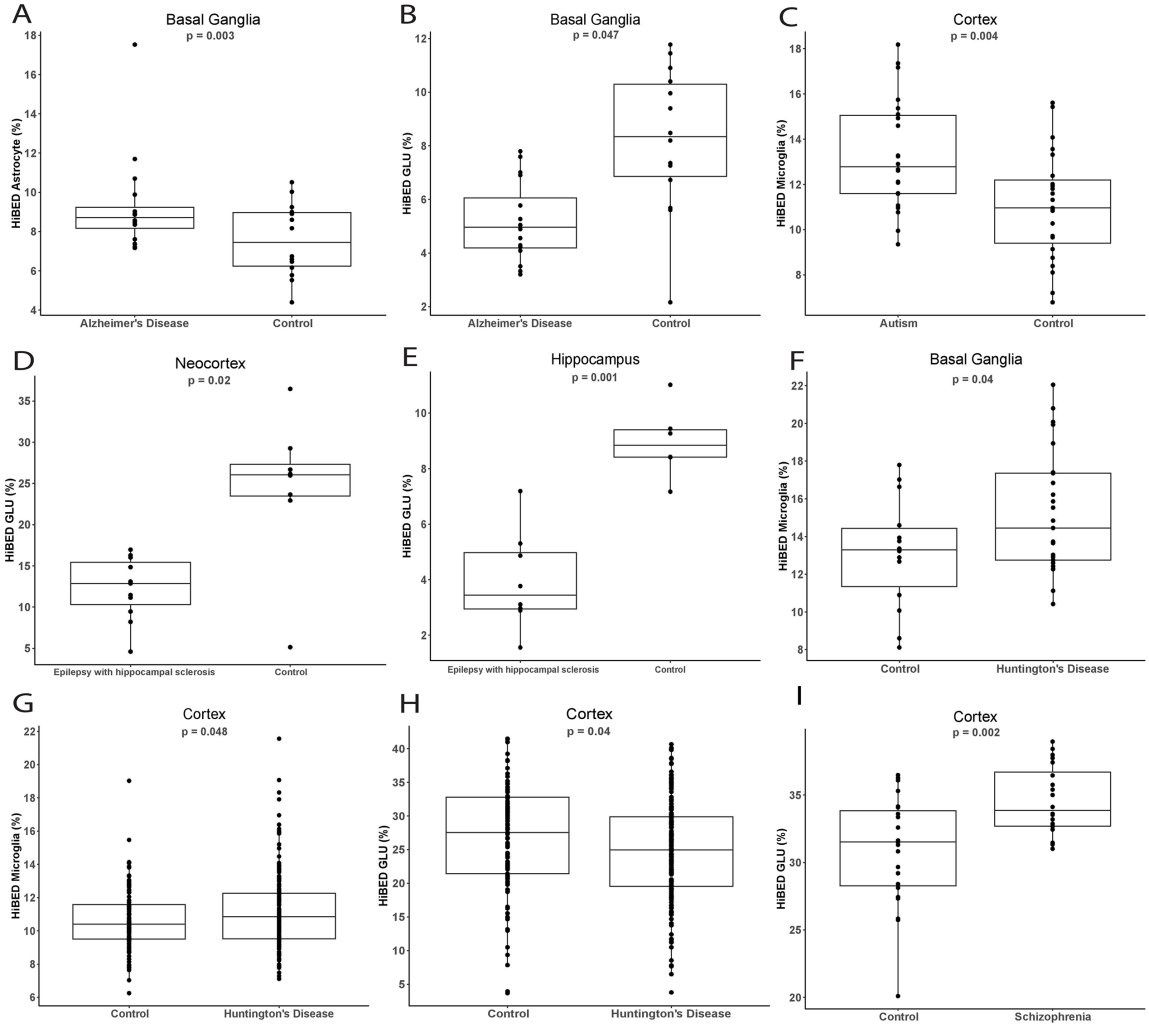
Comparisons of HiBED-predicted cell proportions between cases and normal controls in **(A,B)** Alzheimer’s disease, **(C)** autism, **(D,E)** epilepsy, **(F–H)** Huntington’s disease, **(I)** and schizophrenia across different brain regions..

To probe cell proportion variation in brain-related disorders, we applied the algorithm to independent data sets from GEO to investigate the cellular differential between disease and control samples (Table 4). By comparing basal ganglia DNA methylation data in Alzheimer’s disease patients (n = 16) and controls (*n* = 14) from GSE72778 (Horvath et al., 2016), we observed a significantly higher proportion of astrocyte (Δ = 3.83%, *p* = 0.003, Figure 6A) and a lower proportion of GLU (Δ = 2.53%, *p* = 0.047, Figure 6B) in Alzheimer’s disease patients compared to control samples, adjusting for age and sex. Using cortex methylation data in Autism patients (*n* = 22) and controls (*n* = 23) from GSE53924 (Nardone et al., 2014), we observed a significantly higher proportion of microglia in Autism patients (Δ = 2.14%, *p* = 0.004, Figure 6C) compared to control samples, adjusting for Horvath methylation age. For epilepsy patients (*n* = 19) and control samples (*n* = 14) in the cortex and hippocampus regions from GSE168916 (Martins-Ferreira et al., 2022), we observed significantly lower proportions of GLU in the cortex (Δ = 14.98%, *p* = 0.02, Figure 6D) and hippocampus (Δ = 6.04%, p = 0.001, Figure 6E), adjusting for Horvath methylation age and sex. With methylation data on Huntington’s disease patients (*n* = 197) and controls (*n* = 119) in the region of basal ganglia and cortex from GSE72778 (Horvath et al., 2016), we observed significantly higher proportions of microglia in basal ganglia (Δ = 2.25%, *p* = 0.04, Figure 6F) and cortex (Δ = 0.54%, *p* = 0.048, Figure 6G). A lower proportion of GLU in the cortex (Δ = 2.00%, *p* = 0.04, Figure 6H) for Huntington’s disease patients compared to control samples, adjusting for age and sex. With methylation data on Schizophrenia patients (*n* = 20) and control samples (*n* = 23) in cortex from GSE61431 (Nardone et al., 2014), we observed a significant increase of GLU proportion in Schizophrenia patients compared to control samples (Δ = 3.55%, *p* = 0.002, Figure 6I), adjusting for age and sex. The complete results for each cell type with each condition are shown in Supplementary Table S1. The data demonstrated how HiBED could infer cell type alterations in the pathogenesis of brain-related disorders.

## Discussion

We developed, validated, and applied HiBED, a DNA methylation reference-based method for deconvolving seven brain cell types in bulk brain tissue samples. DNA methylation has been widely used to mark cell fate determination. Initial studies have developed deconvolution methods based on DNA methylation biomarkers to infer the composition of blood leukocytes (Houseman et al., 2012; Salas et al., 2022), cells in the tumor microenvironment (Zhang et al., 2022), fetal origin cells (Salas et al., 2018), and neuronal cells (Guintivano et al., 2013). The current library for CNS sample deconvolution is limited in the number of cell types deconvolved. The CETS method deconvolves the brain cells into NeuN+ and NeuN- cells (Guintivano et al., 2013). The EpiSCORE method expanded the brain cell deconvolution to six cell types but does not include neuronal cell subtypes (Zhu et al., 2022). The human brain has high cellular heterogeneity, and categorizing the mixed population of brain cells with limited cell types provides an incomplete view of the landscape of brain samples. Our deconvolution method extends the utility of brain cell deconvolution to seven major cell types to offer a more comprehensive picture of brain cell heterogeneity.

HiBED was validated by using multiple metrics. We applied the algorithm to the *in silico* synthetic brain cell mixtures with seven cell types, and the deconvolution performed very well. We also validated the method of brain samples used in the CETS package. We confirmed and extended the findings by Guintivano et al. (2013). Although the brain samples in the CETS package only quantified neuronal and non-neuronal cells, the estimation by using our method is consistent with the results from the CETS algorithm. Similar to the CETS algorithm, our method’s deconvolution results showed a higher estimation variation in bulk brain samples than in artificial mixtures. As discussed by Guintivano and colleagues, the variation could be induced at the selection of FANS gate parameters used to define the neuronal population (Guintivano et al., 2013). Also, experimental noise introduced by variation of sectioned bulk tissue samples between FANS and methylation array could cause the observed variation of cell proportion projections between deconvolution and FANS (Guintivano et al., 2013).

Brain regions vary widely by cell constitution, consistent with regional functional purposes. The application of HiBED on standard brain samples in various brain areas showed that our libraries could capture the regional variance of brain cell composition. Fang et al. established 26% excitatory neurons, 11% inhibitory neurons, and 63% non-neuronal cells in the human middle temporal gyrus by using MERFISH (Fang et al., 2022). The results are consistent with HiBED depicted 26.5% excitatory neurons, 7% inhibitory neurons, and 66.5% non-neuronal cells in the human cortex. By depicting the cell type composition, the cerebellum region showed a significantly higher proportion of cells not captured by HiBED while a low level of endothelial cells. The cerebellum is known to be enriched with endothelial cells that are actively engaged in microvascular stasis and leukocyte infiltration (Stanimirovic and Satoh, 2000). The cerebral endothelium is a crucial element of the blood–brain barrier and is deemed a unique type of cell that functions in barrier establishment, regulation of local cerebral blood flow, and interactions with the neurovascular unit (Pretnar-Oblak, 2014; Hainsworth et al., 2015). We hypothesize that the unknown cell proportions in the cerebellum majorly attribute to cerebral endothelium. Although the endothelial cell is included in HiBED, the uniqueness of cerebral endothelium differentiates the cord tissue endothelial cells used in the HiBED reference. Researchers described differential epigenomic landscapes of CNS and non-CNS vascular endothelial cells, contributing to the blood–brain barrier (BBB) differentiation (Sabbagh et al., 2018). As a result, HiBED deconvolution failed to describe endothelium in the cerebellum. Regarding reference-based deconvolution, if a cell type is not well represented in the reference library, it will fall into the remaining unknown cell proportions from deconvolution (Bell-Glenn et al., 2022).

Investigating the relationship between aging and neuronal cells by brain region demonstrated the necessity of adding granularity to brain cell deconvolution. In the human cortex, we observed a negative relationship between neuronal cell proportion and age in males. With our libraries, we could stratify the neuronal cell by subtypes. The stratified results showed that the negative correlation between GABAergic neurons and age primarily drives the negative relationship between neuronal cell proportion and age. The studies of the effect of aging on cortical neurons in the 1970s and 1980s propagated the notion that a significant loss of cortical neurons occurs with normal aging (von Bartheld, 2018). This concept prevailed until Herbert Haug and colleagues concluded that the observed cortical neuron loss with aging was mainly attributed to technical artifacts (Haug et al., 1984). A recent meta-analysis combined data from four stereology studies to investigate the effect of aging on the prevalence of cortical neurons. The study reported a 2–4% decrease in the number of cortical neurons with age in a lifetime (von Bartheld, 2018), consistent with our finding of a 3.75% decrease of neuron proportion in the cortex from age 15 to 108. Previous research established that aging is not associated with massive neuron loss (Wickelgren, 1996), which is consistent with our findings of no significant correlation between age and neuron proportion in the cerebellum, hippocampus, and basal ganglia but a weak negative correlation in the cortex.

However, when stratified by HiBED neuronal subtypes in the cerebellum, a significant loss of GABAergic neuron proportion and gain of glutamatergic neuron proportion was observed. Mouse models reveal that aging-induced impairments of the GABAergic system could lead to an inhibitory/excitatory imbalance, decreasing the neuron’s ability to respond to plastic changes to environmental and cellular challenges (Rozycka and Liguz-Lecznar, 2017). Our findings of decreasing GABAergic neuron proportion and GABAergic/glutamatergic neuron ratio in the cortex and cerebellum support the idea of aging-induced impairments of the GABAergic system. The aging effect on neuronal proportion varied by brain region and neuron subtypes, indicating divergent cellular content alteration by aging in the brain. With the additional layer of neuronal deconvolution, HiBED was able to provide novel insights into cellular variation over the course of aging at a more granular level.

Much effort has been put into quantifying the human brain’s cellular composition over the past century (von Bartheld et al., 2016). However, the research findings could be more consistent and majorly affected by the methods applied (von Bartheld, 2018). The concept of a 10:1 GNR for the entire human brain was standard textbook dogma that was dominant over decades (von Bartheld, 2018). The reports of GNR using different methods varied from 0.7:1 to 50:1 in the past century (von Bartheld, 2018). Major common issues causing technical variation include unclear delineation and definition distinguishing cell types, heterogeneity of sampled tissues, destruction of cells, and technological artifacts.

The isotropic fractionator technique is the most advanced method for determining the cellular composition of nervous tissue to minimize the challenges by labeling the nuclei; however, it could suffer from the destruction of nuclei. The method was applied in recent studies to investigate the GNR in the human cortex. Azevedo et al. reported a 1.05–1.48 GNR in cerebral cortex gray matter, while Andrade-Moraes et al. described a 1.15–1.63 GNR in the same region (Azevedo et al., 2009; Andrade-Moraes et al., 2013). By applying HiBED to normal human cortex DNA methylation data, we observed a mean (SD) GNR of 1.86 (1.15). Another common error occurring when attempting to measure brain cells is to examine one part of the brain and assume the rest of the brain is similarly constituted. In our assessment, brain cellular composition varied substantially by subregion. While the GNR in human cerebral cortex gray matter reported by Azevedo et al. is 1.05–1.48, the GNR is estimated at 2.48 in cerebral cortex gray matter with white matter.

Similarly, in Andrade-Moraes et al. study, the GNR in the human cerebral cortex changed from 1.15 to 1.64 in the gray matter only to 3.01 in gray matter plus white matter. Although our human cortex samples are not distinguished by gray matter or white matter, we observed variations of GNR across major brain subregions. Another metric studied for brain cell counts is the composition of glial cells. Unsurprisingly, the measurement of glial cell composition is also primarily affected by techniques used and specific to brain regions (von Bartheld et al., 2016). The number of glial cell compositions reported varied immensely over the past century — a study by Pelvig et al. in 2008 quantified glial cell composition in the neocortex. Using stereological cell counting, they observed 75% oligodendrocytes, 19% astrocytes, and 6% microglia composing the glial cells (Pelvig et al., 2008). In 2009, Lyck and colleagues improved the method by combining stereological cell counting with immunohistochemical visualization and observed 15–18% microglia contributing to glial cell composition (Lyck et al., 2009). In our study, the DNA methylation quantification revealed cortical glial cell composition with 66.04% (±8.70%) oligodendrocytes, 13.79% (±4.15%)astrocytes, and 20.17% (±5.45%) microglia. Taken together, brain cell quantification is affected mainly by the techniques used. Different challenges associated with the methods employed can cause false results. Thus, no uniform understanding of brain cell composition is established, even to this day. Our deconvolution algorithm utilized differential DNA methylation profiles specific to brain cell subtypes, circumventing some of the significant challenges, including artificial bias in counting the cells and the destruction of the nuclei for estimating the cell proportions.

Brain cell alterations have been studied in various neurological and psychiatric diseases (Stockmeier and Rajkowska, 2004; Stark et al., 2005; Dorph-Petersen et al., 2007; Reiner et al., 2011; Schmaal et al., 2016). Although abnormal glial numbers, neuron numbers, or GNRs were implicated in neurological and psychiatric disorders, studies lack validity and reliability in the quantitative data (Williams and Rakic, 1988; von Bartheld, 2018). Such impediment is majorly derived from the inconsistency of techniques applied to measure brain cells (von Bartheld et al., 2016; von Bartheld, 2018). Using HiBED to investigate neurological and psychiatric disease samples, we have confirmed recent findings on brain cell alteration in Alzheimer’s disease, autism, epilepsy, Huntington’s disease, and schizophrenia. Reactive astrocyte and decreased glutamate levels were observed in Alzheimer’s disease (Fayed et al., 2011; Smit et al., 2021), consistent with significantly increased astrocyte proportion and decreased GLU proportion captured by HiBED in Alzheimer’s patients. In Autism, researchers observed microglial activation and greater microglial densities in cortical areas (Rodriguez and Kern, 2011; Tetreault et al., 2012). Consistently, HiBED illustrated significantly higher microglial cell proportion in Autism patients compared to controls. Glutamatergic mechanisms are associated with epilepsy development (Cho, 2013; Barker-Haliski and White, 2015). In our data, GLU proportion was lower in epilepsy patients compared to normal controls. Microglial activation and reactivity are known to play a crucial role in the pathogenesis of Huntington’s disease (Yang et al., 2017; Savage et al., 2020), which is consistent with elevated microglia proportion estimated by HiBED in Huntington’s disease patients compared to control samples.

Furthermore, Andre et al. observed a decrease of glutamate neurotransmission as Huntington’s disease phenotype develops, which aligns with the lower level of GLU proportion captured by HiBED in Huntington’s disease patients compared to normal controls (Andre et al., 2010). Finally, the glutamate hypothesis of schizophrenia was proposed by Moghaddam et al. attributed the symptoms and cognitive impairment to hypofunction of NMDA receptors and excessive glutamate release, especially in the prefrontal cortex (Moghaddam and Javitt, 2012). In our study, HiBED described a significant increase in GLU proportion in schizophrenia patients compared to normal controls.

While the results strongly suggest that HiBED is a valid method for estimating significant cell proportions in CNS samples with many potential applications to brain-related research, we also recognize some limitations. Firstly, due to the challenges of isolating and identifying publicly available DNA methylation data for brain endothelial cells and stromal cells, we used cord tissue endothelial cells and cord tissue stromal cells as proxies for brain-specific endothelial and stromal cells. From an ontogenesis perspective, significant epigenomic similarities between those cell types and their counterparts are expectedly shared across different tissue types. We, therefore, posited a reasonable representation of endothelial and stromal cells in the library. However, we hypothesize that the cerebral endothelium is unique and distinguished from HiBED-predicted endothelial cells. Thus, future studies would be ideal to add cerebral endothelium into the reference library. Secondly, although seven major brain cell types are included in the libraries, representing the true brain cells’ heterogeneity demands a more granular library. For neuron cells, our library delineated the excitatory neuron from the inhibitory neuron. However, a recent single-nucleus RNA sequencing study identified 16 neuronal subtypes within the excitatory and inhibitory neurons (Lake et al., 2016). Furthermore, a mouse model by Marques et al. in 2016 identified 12 subpopulations of oligodendrocytes (Marques et al., 2016), but our current libraries need to distinguish oligodendrocyte subtypes. Other potential cell subtypes that could be added to the libraries are infiltrating leukocytes. Brain leukocyte infiltration is initiated by neuroinflammation (Schmitt et al., 2012). Thus, CNS samples with neurological diseases would likely contain infiltrating leukocytes. The brain infiltrating leukocytes are not included in the libraries because of the challenges of isolating and identifying them. Using overloading proxies would generate noise that likely would bias any estimation. Thirdly, the libraries are mainly validated using *in silico* synthetic mixture samples generated from the discovery data set. The external validation data sets do not cover all brain cell subtypes in the libraries. Thus, a comprehensive external validation of all cell type proportion projections is not feasible in this study. However, the validation results from the artificial mixtures and the external validation data sets provided strong evidence of the method’s validity.

Future studies aiming to extend the library to comprehend brain cell heterogeneity better will be necessary to refine the deconvolution model. With the brain cell complexity gradually unveiled by single-cell sequencing technology and the advance of cell isolation and purification procedure, more brain cell subtypes can be added to the library. For HiBED’s application, the deconvolution in EWAS in brain-related research would help identify differentially methylated regions independent of cell population confounding. Furthermore, applying the method to neurological and psychiatric DNA methylation analysis can reveal novel discoveries related to pathobiological brain cell alteration and epigenetic regulation of the disease.

## Conclusion

We developed, validated, and applied a DNA methylation-based brain cell deconvolution method, HiBED, designed to infer the proportions of seven major brain cell types. HiBED employed a hierarchical deconvolution approach with optimized libraries for neuronal, glial, and endothelial and stromal cells. HiBED provides higher cell type resolution compared to existing methods for brain deconvolution, enabling new opportunities to study cell heterogeneity in brain-related diseases and potential therapeutic targets.

## Data availability statement

The data sets used in this study are available on NCBI Gene Expression Omnibus (GEO, https://www.ncbi.nlm.nih.gov/geo/) with accession numbers GSE40360, GSE61380, GSE43414, GSE61431, GSE72778, GSE110554, GSE107729, GSE134165, GSE61380, GSE30339, GSE41826, GSE61431, GSE66351, GSE72778, GSE76105, GSE80970, GSE88890, GSE89703, GSE43414, and GSE79064 and SYNAPSE (https://www.synapse.org/) with accession number syn4588488. The HiBED package is publicly available at https://github.com/SalasLab/HiBED.

## Author contributions

ZZ, LS, JW, KK, DK, AM, and BC proposed the original idea. ZZ, LS, SP, and PK contributed to the processing and bioinformatic analyses of the paper. ZZ, JW, KK, DK, AM, SP, PK, BC, and LS participated in the interpretation of data for the work and participated in the final drafting and critical revision of important intellectual content. ZZ and LS were responsible for the initial draft of the work. All authors contributed to the article and approved the submitted version.

## Funding

This work was supported by R01CA253976, R01CA216265, P30CA023108, W81XWH-20-1-0778, P20GM104416/8299, P20GM130423, P30CA168524, P20GM103418, P50CA097257, R01CA207360, and 4K00CA264400–02 and the Robert Magnin Newman Endowed Chair in Neuro-oncology.

## Conflict of interest

The authors declare that the research was conducted in the absence of any commercial or financial relationships that could be construed as a potential conflict of interest.

## Publisher’s note

All claims expressed in this article are solely those of the authors and do not necessarily represent those of their affiliated organizations, or those of the publisher, the editors and the reviewers. Any product that may be evaluated in this article, or claim that may be made by its manufacturer, is not guaranteed or endorsed by the publisher.

## Footnotes

^1^https://github.com/SalasLab/HiBED

## Abbreviations

HiBED: hierarchical brain extended deconvolution
WGBS: whole-genome bisulfite sequencing
sc-RNA: single-cell RNA
NeuN: neuronal nuclei
GABA: GABAergic neurons
GLU: glutamatergic neurons
CETS: cell epigenotype specific
GNR: glia to neuron ratio
CNS: central nervous system
CP/QP: constrained projection/ quadratic programming
EWAS: epigenome-wide association studies
FACS: fluorescence-activated cell sorting
FANS: fluorescence-activated nuclei sorting
Pearson’s r: Pearson’s correlation coefficient
SD: standard deviation
R2: coefficient of determination
RMSE: root mean square error

## References

[ref1] An X. Chen L. (2018). Flow cytometry (FCM) analysis and fluorescence-activated cell sorting (FACS) of erythroid cells. Methods Mol. Biol. 1698, 153–174. doi: 10.1007/978-1-4939-7428-3_9, PMID: 29076089

[ref2] Andrade-Moraes C. H. Oliveira-Pinto A. V. Castro-Fonseca E. da Silva C. G. Guimaraes D. M. Szczupak D.. (2013). Cell number changes in Alzheimer's disease relate to dementia, not to plaques and tangles. Brain 136, 3738–3752. doi: 10.1093/brain/awt273, PMID: 24136825 PMC3859218

[ref3] Andre V. M. Cepeda C. Levine M. S. (2010). Dopamine and glutamate in Huntington's disease: a balancing act. CNS Neurosci. Ther. 16, 163–178. doi: 10.1111/j.1755-5949.2010.00134.x, PMID: 20406248 PMC3118459

[ref4] Azevedo F. A. Carvalho L. R. Grinberg L. T. Farfel J. M. Ferretti R. E. Leite R. E.. (2009). Equal numbers of neuronal and nonneuronal cells make the human brain an isometrically scaled-up primate brain. J. Comp. Neurol. 513, 532–541. doi: 10.1002/cne.21974, PMID: 19226510

[ref5] Barker-Haliski M. White H. S. (2015). Glutamatergic mechanisms associated with seizures and epilepsy. Cold Spring Harb. Perspect. Med. 5:a022863. doi: 10.1101/cshperspect.a022863, PMID: 26101204 PMC4526718

[ref6] Bell-Glenn S. Thompson J. A. Salas L. A. Koestler D. C. (2022). A novel framework for the identification of reference DNA methylation libraries for reference-based deconvolution of cellular mixtures. Front Bioinform. 2:2. doi: 10.3389/fbinf.2022.835591 PMC9004796 35419567

[ref7] Bogdanovic O. Lister R. (2017). DNA methylation and the preservation of cell identity. Curr. Opin. Genet. Dev. 46, 9–14. doi: 10.1016/j.gde.2017.06.007, PMID: 28651214

[ref8] Cho C. H. (2013). New mechanism for glutamate hypothesis in epilepsy. Front. Cell. Neurosci. 7:127. doi: 10.3389/fncel.2013.00127 23964202 PMC3741557

[ref9] Cossarizza A. Chang H. D. Radbruch A. Akdis M. Andra I. Annunziato F.. (2017). Guidelines for the use of flow cytometry and cell sorting in immunological studies. Eur. J. Immunol. 47, 1584–1797. doi: 10.1002/eji.201646632, PMID: 29023707 PMC9165548

[ref10] Crouch E. E. Doetsch F. (2018). FACS isolation of endothelial cells and pericytes from mouse brain microregions. Nat. Protoc. 13, 738–751. doi: 10.1038/nprot.2017.158, PMID: 29565899

[ref11] De Souza R. A. Islam S. A. McEwen L. M. Mathelier A. Hill A. Mah S. M.. (2016). DNA methylation profiling in human Huntington's disease brain. Hum. Mol. Genet. 25, 2013–2030. doi: 10.1093/hmg/ddw076, PMID: 26953320

[ref12] de Witte L. D. Wang Z. Snijders G. Mendelev N. Liu Q. Sneeboer M. A. M.. (2022). Contribution of age, brain region, mood disorder pathology, and Interindividual factors on the Methylome of human microglia. Biol. Psychiatry 91, 572–581. doi: 10.1016/j.biopsych.2021.10.020, PMID: 35027166 PMC11181298

[ref13] Dorph-Petersen K. A. Pierri J. N. Wu Q. Sampson A. R. Lewis D. A. (2007). Primary visual cortex volume and total neuron number are reduced in schizophrenia. J. Comp. Neurol. 501, 290–301. doi: 10.1002/cne.21243, PMID: 17226750

[ref14] Egusa H. Iida K. Kobayashi M. Lin T. Y. Zhu M. Zuk P. A.. (2007). Downregulation of extracellular matrix-related gene clusters during osteogenic differentiation of human bone marrow- and adipose tissue-derived stromal cells. Tissue Eng. 13, 2589–2600. doi: 10.1089/ten.2007.0080, PMID: 17666000

[ref15] Fang R. Xia C. Close J. L. Zhang M. He J. Huang Z.. (2022). Conservation and divergence of cortical cell organization in human and mouse revealed by MERFISH. Science 377, 56–62. doi: 10.1126/science.abm1741, PMID: 35771910 PMC9262715

[ref16] Fayed N. Modrego P. J. Rojas-Salinas G. Aguilar K. (2011). Brain glutamate levels are decreased in Alzheimer's disease: a magnetic resonance spectroscopy study. Am. J. Alzheimers Dis. Other Dement. 26, 450–456. doi: 10.1177/1533317511421780 PMC10845671 21921084

[ref17] Gasparoni G. Bultmann S. Lutsik P. Kraus T. F. J. Sordon S. Vlcek J.. (2018). DNA methylation analysis on purified neurons and glia dissects age and Alzheimer's disease-specific changes in the human cortex. Epigenetics Chromatin 11:41. doi: 10.1186/s13072-018-0211-3, PMID: 30045751 PMC6058387

[ref18] Goyette S. R. Schott E. Uwimana A. Nelson D. W. Boganski J. (2019). Detection of the steroid receptor interacting protein, PAK6, in a neuronal cell line. Heliyon 5:e01294. doi: 10.1016/j.heliyon.2019.e01294, PMID: 30923762 PMC6423815

[ref19] Guez-Barber D. Fanous S. Harvey B. K. Zhang Y. Lehrmann E. Becker K. G.. (2012). FACS purification of immunolabeled cell types from adult rat brain. J. Neurosci. Methods 203, 10–18. doi: 10.1016/j.jneumeth.2011.08.045, PMID: 21911005 PMC3221768

[ref20] Guillaumet-Adkins A. Heyn H. (2017). Single-cell genomics unravels brain cell-type complexity. Adv. Exp. Med. Biol. 978, 393–407. doi: 10.1007/978-3-319-53889-1_20, PMID: 28523557

[ref21] Guintivano J. Aryee M. J. Kaminsky Z. A. (2013). A cell epigenotype specific model for the correction of brain cellular heterogeneity bias and its application to age, brain region and major depression. Epigenetics 8, 290–302. doi: 10.4161/epi.23924, PMID: 23426267 PMC3669121

[ref22] Gusel'nikova V. V. Korzhevskiy D. E. (2015). NeuN as a neuronal nuclear antigen and neuron differentiation marker. Acta Nat. 7, 42–47. doi: 10.32607/20758251-2015-7-2-42-47 PMC4463411 26085943

[ref23] Hainsworth A. H. Oommen A. T. Bridges L. R. (2015). Endothelial cells and human cerebral small vessel disease. Brain Pathol. 25, 44–50. doi: 10.1111/bpa.12224, PMID: 25521176 PMC8029339

[ref24] Haug H. Kuhl S. Mecke E. Sass N. L. Wasner K. (1984). The significance of morphometric procedures in the investigation of age changes in cytoarchitectonic structures of human brain. J. Hirnforsch. 25, 353–374. PMID: 6481152

[ref25] Herbomel P. Thisse B. Thisse C. (1999). Ontogeny and behaviour of early macrophages in the zebrafish embryo. Development 126, 3735–3745. doi: 10.1242/dev.126.17.3735, PMID: 10433904

[ref26] Horvath S. Langfelder P. Kwak S. Aaronson J. Rosinski J. Vogt T. F.. (2016). Huntington's disease accelerates epigenetic aging of human brain and disrupts DNA methylation levels. Aging (Albany NY) 8, 1485–1512. doi: 10.18632/aging.101005, PMID: 27479945 PMC4993344

[ref27] Houseman E. A. Accomando W. P. Koestler D. C. Christensen B. C. Marsit C. J. Nelson H. H.. (2012). DNA methylation arrays as surrogate measures of cell mixture distribution. BMC Bioinformatics. 13:86. doi: 10.1186/1471-2105-13-86, PMID: 22568884 PMC3532182

[ref28] Jin W. Dai Y. Li F. Zhu L. Huang Z. Liu W.. (2019). Dysregulation of microglial function contributes to neuronal impairment in Mcoln1a-deficient zebrafish. iScience 13, 391–401. doi: 10.1016/j.isci.2019.02.031, PMID: 30897512 PMC6426713

[ref29] Kent W. J. Sugnet C. W. Furey T. S. Roskin K. M. Pringle T. H. Zahler A. M.. (2002). The human genome browser at UCSC. Genome Res. 12, 996–1006. doi: 10.1101/gr.229102, PMID: 12045153 PMC186604

[ref30] Koestler D. C. Jones M. J. Usset J. Christensen B. C. Butler R. A. Kobor M. S.. (2016). Improving cell mixture deconvolution by identifying optimal DNA methylation libraries (IDOL). BMC Bioinformatics. 17:120. doi: 10.1186/s12859-016-0943-7, PMID: 26956433 PMC4782368

[ref31] Kozlenkov A. Li J. Apontes P. Hurd Y. L. Byne W. M. Koonin E. V.. (2018). A unique role for DNA (hydroxy)methylation in epigenetic regulation of human inhibitory neurons. Sci. Adv. 4:eaau6190. doi: 10.1126/sciadv.aau6190 30263963 PMC6157969

[ref32] Lake B. B. Ai R. Kaeser G. E. Salathia N. S. Yung Y. C. Liu R.. (2016). Neuronal subtypes and diversity revealed by single-nucleus RNA sequencing of the human brain. Science 352, 1586–1590. doi: 10.1126/science.aaf1204, PMID: 27339989 PMC5038589

[ref33] Leavitt T. Hu M. S. Longaker M. T. (2017). Isolation of live fibroblasts by fluorescence-activated cell sorting. Methods Mol. Biol. 1627, 205–212. doi: 10.1007/978-1-4939-7113-8_13, PMID: 28836203

[ref34] Lin X. Tan J. Y. L. Teh A. L. Lim I. Y. Liew S. J. MacIsaac J. L.. (2018). Cell type-specific DNA methylation in neonatal cord tissue and cord blood: a 850K-reference panel and comparison of cell types. Epigenetics 13, 941–958. doi: 10.1080/15592294.2018.1522929, PMID: 30232931 PMC6284779

[ref35] Lu J. Li C. Shi C. Balducci J. Huang H. Ji H. L.. (2012). Identification of novel splice variants and exons of human endothelial cell-specific chemotaxic regulator (ECSCR) by bioinformatics analysis. Comput. Biol. Chem. 41, 41–50. doi: 10.1016/j.compbiolchem.2012.10.003, PMID: 23147565 PMC3513626

[ref36] Lyck L. Santamaria I. D. Pakkenberg B. Chemnitz J. Schroder H. D. Finsen B.. (2009). An empirical analysis of the precision of estimating the numbers of neurons and glia in human neocortex using a fractionator-design with sub-sampling. J. Neurosci. Methods 182, 143–156. doi: 10.1016/j.jneumeth.2009.06.003, PMID: 19520115

[ref37] Marcilla A. Bargues M. D. Ramsey J. M. Magallon-Gastelum E. Salazar-Schettino P. M. Abad-Franch F.. (2001). The ITS-2 of the nuclear rDNA as a molecular marker for populations, species, and phylogenetic relationships in Triatominae (Hemiptera: Reduviidae), vectors of Chagas disease. Mol. Phylogenet. Evol. 18, 136–142. doi: 10.1006/mpev.2000.0864, PMID: 11161750

[ref38] Marques S. Zeisel A. Codeluppi S. van Bruggen D. Mendanha Falcao A. Xiao L.. (2016). Oligodendrocyte heterogeneity in the mouse juvenile and adult central nervous system. Science 352, 1326–1329. doi: 10.1126/science.aaf6463, PMID: 27284195 PMC5221728

[ref39] Martins-Ferreira R. Leal B. Chaves J. Li T. Ciudad L. Rangel R.. (2022). Epilepsy progression is associated with cumulative DNA methylation changes in inflammatory genes. Prog. Neurobiol. 209:102207. doi: 10.1016/j.pneurobio.2021.102207, PMID: 34923048

[ref40] Mendizabal I. Berto S. Usui N. Toriumi K. Chatterjee P. Douglas C.. (2019). Cell type-specific epigenetic links to schizophrenia risk in the brain. Genome Biol. 20:135. doi: 10.1186/s13059-019-1747-7, PMID: 31288836 PMC6617737

[ref41] Milward K. Hester J. Wood K. J. (2019). Isolation of human regulatory T lymphocytes by fluorescence-activated cell sorting. Methods Mol. Biol. 1899, 43–54. doi: 10.1007/978-1-4939-8938-6_4 30649764

[ref42] Min J. L. Hemani G. Davey Smith G. Relton C. Suderman M. (2018). Meffil: efficient normalization and analysis of very large DNA methylation datasets. Bioinformatics 34, 3983–3989. doi: 10.1093/bioinformatics/bty476, PMID: 29931280 PMC6247925

[ref43] Moghaddam B. Javitt D. (2012). From revolution to evolution: the glutamate hypothesis of schizophrenia and its implication for treatment. Neuropsychopharmacology 37, 4–15. doi: 10.1038/npp.2011.181, PMID: 21956446 PMC3238069

[ref44] Mu Q. Chen Y. Wang J. (2019). Deciphering brain complexity using single-cell sequencing. Genomics Proteomics Bioinformatics 17, 344–366. doi: 10.1016/j.gpb.2018.07.007, PMID: 31586689 PMC6943771

[ref45] Murphy T. M. Crawford B. Dempster E. L. Hannon E. Burrage J. Turecki G.. (2017). Methylomic profiling of cortex samples from completed suicide cases implicates a role for PSORS1C3 in major depression and suicide. Transl. Psychiatry 7:e989. doi: 10.1038/tp.2016.249, PMID: 28045465 PMC5545719

[ref46] Muse M. E. Bergman D. T. Salas L. A. Tom L. N. Tan J. M. Laino A.. (2022). Genome-scale DNA methylation analysis identifies repeat element alterations that modulate the genomic stability of melanocytic nevi. J. Invest. Dermatol. 142, 1893–1902.e7. doi: 10.1016/j.jid.2021.11.025, PMID: 34871578 PMC9163203

[ref47] Muse M. E. Carroll C. D. Salas L. A. Karagas M. R. Christensen B. C. (2023). Application of novel breast biospecimen cell type adjustment identifies shared DNA methylation alterations in breast tissue and milk with breast cancer risk factors. Cancer Epidemiol. Biomark. Prev. 32, 550–560. doi: 10.1158/1055-9965.EPI-22-0405, PMID: 36780234 PMC10068446

[ref48] Nardone S. Sams D. S. Reuveni E. Getselter D. Oron O. Karpuj M.. (2014). DNA methylation analysis of the autistic brain reveals multiple dysregulated biological pathways. Transl. Psychiatry 4:e433. doi: 10.1038/tp.2014.70, PMID: 25180572 PMC4203003

[ref49] Pelvig D. P. Pakkenberg H. Stark A. K. Pakkenberg B. (2008). Neocortical glial cell numbers in human brains. Neurobiol. Aging 29, 1754–1762. doi: 10.1016/j.neurobiolaging.2007.04.013 17544173

[ref50] Pidsley R. Cc Y. W. Volta M. Lunnon K. Mill J. Schalkwyk L. C. (2013). A data-driven approach to preprocessing Illumina 450K methylation array data. BMC Genomics 14:293. doi: 10.1186/1471-2164-14-293, PMID: 23631413 PMC3769145

[ref51] Pidsley R. Viana J. Hannon E. Spiers H. Troakes C. Al-Saraj S.. (2014). Methylomic profiling of human brain tissue supports a neurodevelopmental origin for schizophrenia. Genome Biol. 15:483. doi: 10.1186/s13059-014-0483-2, PMID: 25347937 PMC4262979

[ref52] Pretnar-Oblak J. (2014). Cerebral endothelial function determined by cerebrovascular reactivity to L-arginine. Biomed. Res. Int. 2014:601515, 1–8. doi: 10.1155/2014/601515 24860826 PMC4016874

[ref53] Reiner A. Dragatsis I. Dietrich P. (2011). Genetics and neuropathology of Huntington's disease. Int. Rev. Neurobiol. 98, 325–372. doi: 10.1016/B978-0-12-381328-2.00014-6, PMID: 21907094 PMC4458347

[ref54] Rizzardi L. F. Hickey P. F. Rodriguez DiBlasi V. Tryggvadottir R. Callahan C. M. Idrizi A.. (2019). Neuronal brain-region-specific DNA methylation and chromatin accessibility are associated with neuropsychiatric trait heritability. Nat. Neurosci. 22, 307–316. doi: 10.1038/s41593-018-0297-8, PMID: 30643296 PMC6348048

[ref55] Rodriguez J. I. Kern J. K. (2011). Evidence of microglial activation in autism and its possible role in brain underconnectivity. Neuron Glia Biol. 7, 205–213. doi: 10.1017/S1740925X12000142, PMID: 22874006 PMC3523548

[ref56] Rozycka A. Liguz-Lecznar M. (2017). The space where aging acts: focus on the GABAergic synapse. Aging Cell 16, 634–643. doi: 10.1111/acel.12605, PMID: 28497576 PMC5506442

[ref57] Sabbagh M. F. Heng J. S. Luo C. Castanon R. G. Nery J. R. Rattner A.. (2018). Transcriptional and epigenomic landscapes of CNS and non-CNS vascular endothelial cells. elife 7:7. doi: 10.7554/eLife.36187 PMC6126923 30188322

[ref58] Salas L. A. Koestler D. C. Butler R. A. Hansen H. M. Wiencke J. K. Kelsey K. T.. (2018). An optimized library for reference-based deconvolution of whole-blood biospecimens assayed using the Illumina HumanMethylationEPIC BeadArray. Genome Biol. 19:64. doi: 10.1186/s13059-018-1448-7, PMID: 29843789 PMC5975716

[ref59] Salas L. A. Wiencke J. K. Koestler D. C. Zhang Z. Christensen B. C. Kelsey K. T. (2018). Tracing human stem cell lineage during development using DNA methylation. Genome Res. 28, 1285–1295. doi: 10.1101/gr.233213.117, PMID: 30072366 PMC6120629

[ref60] Salas L. A. Zhang Z. Koestler D. C. Butler R. A. Hansen H. M. Molinaro A. M.. (2022). Enhanced cell deconvolution of peripheral blood using DNA methylation for high-resolution immune profiling. Nat. Commun. 13:761. doi: 10.1038/s41467-021-27864-7, PMID: 35140201 PMC8828780

[ref61] Sarkar T. J. Quarta M. Mukherjee S. Colville A. Paine P. Doan L.. (2020). Transient non-integrative expression of nuclear reprogramming factors promotes multifaceted amelioration of aging in human cells. Nat. Commun. 11:1545. doi: 10.1038/s41467-020-15174-3, PMID: 32210226 PMC7093390

[ref62] Savage J. C. St-Pierre M. K. Carrier M. El Hajj H. Novak S. W. Sanchez M. G.. (2020). Microglial physiological properties and interactions with synapses are altered at presymptomatic stages in a mouse model of Huntington's disease pathology. J. Neuroinflammation 17:98. doi: 10.1186/s12974-020-01782-9, PMID: 32241286 PMC7118932

[ref63] Schmaal L. Veltman D. J. van Erp T. G. Samann P. G. Frodl T. Jahanshad N.. (2016). Subcortical brain alterations in major depressive disorder: findings from the ENIGMA major depressive disorder working group. Mol. Psychiatry 21, 806–812. doi: 10.1038/mp.2015.69, PMID: 26122586 PMC4879183

[ref64] Schmitt C. Strazielle N. Ghersi-Egea J. F. (2012). Brain leukocyte infiltration initiated by peripheral inflammation or experimental autoimmune encephalomyelitis occurs through pathways connected to the CSF-filled compartments of the forebrain and midbrain. J. Neuroinflammation 9:187. doi: 10.1186/1742-2094-9-187, PMID: 22870891 PMC3458946

[ref65] Serrano-Pozo A. Frosch M. P. Masliah E. Hyman B. T. (2011). Neuropathological alterations in Alzheimer disease. Cold Spring Harb. Perspect. Med. 1:a006189. doi: 10.1101/cshperspect.a006189, PMID: 22229116 PMC3234452

[ref66] Shi Y. Chichung Lie D. Taupin P. Nakashima K. Ray J. Yu R. T.. (2004). Expression and function of orphan nuclear receptor TLX in adult neural stem cells. Nature 427, 78–83. doi: 10.1038/nature02211 14702088

[ref67] Smit T. Deshayes N. A. C. Borchelt D. R. Kamphuis W. Middeldorp J. Hol E. M. (2021). Reactive astrocytes as treatment targets in Alzheimer's disease-systematic review of studies using the APPswePS1dE9 mouse model. Glia 69, 1852–1881. doi: 10.1002/glia.23981, PMID: 33634529 PMC8247905

[ref68] Smith R. G. Hannon E. De Jager P. L. Chibnik L. Lott S. J. Condliffe D.. (2018). Elevated DNA methylation across a 48-kb region spanning the HOXA gene cluster is associated with Alzheimer's disease neuropathology. Alzheimers Dement. 14, 1580–1588. doi: 10.1016/j.jalz.2018.01.017, PMID: 29550519 PMC6438205

[ref69] Stanimirovic D. Satoh K. (2000). Inflammatory mediators of cerebral endothelium: a role in ischemic brain inflammation. Brain Pathol. 10, 113–126. doi: 10.1111/j.1750-3639.2000.tb00248.x, PMID: 10668901 PMC8098501

[ref70] Stark A. K. Pelvig D. P. Jorgensen A. M. Andersen B. B. Pakkenberg B. (2005). Measuring morphological and cellular changes in Alzheimer's dementia: a review emphasizing stereology. Curr. Alzheimer Res. 2, 449–481. doi: 10.2174/156720505774330528, PMID: 16248850

[ref71] Stockmeier C. A. Rajkowska G. (2004). Cellular abnormalities in depression: evidence from postmortem brain tissue. Dialogues Clin. Neurosci. 6, 185–197. doi: 10.31887/DCNS.2004.6.2/cstockmeier, PMID: 22033633 PMC3181793

[ref72] Suarez-Pinilla M. Fernandez-Vega I. (2015). An acute metabolic insult highly increased postmortem cerebellar autolysis: an autopsy case. Clin. Neuropathol. 34, 166–168. doi: 10.5414/NP300809, PMID: 25492888

[ref73] Teschendorff A. E. Marabita F. Lechner M. Bartlett T. Tegner J. Gomez-Cabrero D.. (2013). A beta-mixture quantile normalization method for correcting probe design bias in Illumina Infinium 450 k DNA methylation data. Bioinformatics 29, 189–196. doi: 10.1093/bioinformatics/bts680, PMID: 23175756 PMC3546795

[ref74] Teschendorff A. E. Zhu T. Breeze C. E. Beck S. (2020). EPISCORE: cell type deconvolution of bulk tissue DNA methylomes from single-cell RNA-Seq data. Genome Biol. 21:221. doi: 10.1186/s13059-020-02126-9, PMID: 32883324 PMC7650528

[ref75] Tetreault N. A. Hakeem A. Y. Jiang S. Williams B. A. Allman E. Wold B. J.. (2012). Microglia in the cerebral cortex in autism. J. Autism Dev. Disord. 42, 2569–2584. doi: 10.1007/s10803-012-1513-0 22466688

[ref76] Tian Y. Morris T. J. Webster A. P. Yang Z. Beck S. Feber A.. (2017). ChAMP: updated methylation analysis pipeline for Illumina BeadChips. Bioinformatics 33, 3982–3984. doi: 10.1093/bioinformatics/btx513, PMID: 28961746 PMC5860089

[ref77] Titus A. J. Gallimore R. M. Salas L. A. Christensen B. C. (2017). Cell-type deconvolution from DNA methylation: a review of recent applications. Hum. Mol. Genet. 26, R216–R224. doi: 10.1093/hmg/ddx275, PMID: 28977446 PMC5886462

[ref78] Titus A. J. Houseman E. A. Johnson K. C. Christensen B. C. (2016). methyLiftover: cross-platform DNA methylation data integration. Bioinformatics 32, 2517–2519. doi: 10.1093/bioinformatics/btw180, PMID: 27153617 PMC5006233

[ref79] Tripathi R. Aggarwal T. Lindberg F. A. Klemm A. H. Fredriksson R. (2022). SLC38A10 regulate glutamate homeostasis and modulate the AKT/TSC2/mTOR pathway in mouse primary cortex cells. Front. Cell Dev. Biol. 10:854397. doi: 10.3389/fcell.2022.854397, PMID: 35450293 PMC9017388

[ref80] Tsuchiya A. Sakamoto M. Yasuda J. Chuma M. Ohta T. Ohki M.. (2003). Expression profiling in ovarian clear cell carcinoma: identification of hepatocyte nuclear factor-1 beta as a molecular marker and a possible molecular target for therapy of ovarian clear cell carcinoma. Am. J. Pathol. 163, 2503–2512. doi: 10.1016/S0002-9440(10)63605-X, PMID: 14633622 PMC1892387

[ref81] Uhlen M. Fagerberg L. Hallstrom B. M. Lindskog C. Oksvold P. Mardinoglu A.. (2015). Proteomics. Tissue-based map of the human proteome. Science 347:1260419. doi: 10.1126/science.1260419 25613900

[ref82] Viana J. Hannon E. Dempster E. Pidsley R. Macdonald R. Knox O.. (2017). Schizophrenia-associated methylomic variation: molecular signatures of disease and polygenic risk burden across multiple brain regions. Hum. Mol. Genet. 26, 210–225. doi: 10.1093/hmg/ddw373, PMID: 28011714 PMC5351932

[ref83] von Bartheld C. S. (2018). Myths and truths about the cellular composition of the human brain: a review of influential concepts. J. Chem. Neuroanat. 93, 2–15. doi: 10.1016/j.jchemneu.2017.08.004, PMID: 28873338 PMC5834348

[ref84] von Bartheld C. S. Bahney J. Herculano-Houzel S. (2016). The search for true numbers of neurons and glial cells in the human brain: a review of 150 years of cell counting. J. Comp. Neurol. 524, 3865–3895. doi: 10.1002/cne.24040, PMID: 27187682 PMC5063692

[ref85] Watson C. T. Roussos P. Garg P. Ho D. J. Azam N. Katsel P. L.. (2016). Genome-wide DNA methylation profiling in the superior temporal gyrus reveals epigenetic signatures associated with Alzheimer's disease. Genome Med. 8:5. doi: 10.1186/s13073-015-0258-8, PMID: 26803900 PMC4719699

[ref86] Weightman Potter P. G. Washer S. J. Jeffries A. R. Holley J. E. Gutowski N. J. Dempster E. L.. (2021). Attenuated induction of the unfolded protein response in adult human primary astrocytes in response to recurrent low glucose. Front Endocrinol (Lausanne). 12:671724. doi: 10.3389/fendo.2021.671724, PMID: 34122346 PMC8187939

[ref87] Wickelgren I. (1996). For the cortex, neuron loss may be less than thought. Science 273, 48–50. doi: 10.1126/science.273.5271.48 8658193

[ref88] Williams R. W. Rakic P. (1988). Three-dimensional counting: an accurate and direct method to estimate numbers of cells in sectioned material. J. Comp. Neurol. 278, 344–352. doi: 10.1002/cne.902780305, PMID: 3216047

[ref89] Wu S. Nguyen L. T. M. Pan H. Hassan S. Dai Y. Xu J.. (2020). Two phenotypically and functionally distinct microglial populations in adult zebrafish. Sci. Adv. 6:eabd1160. doi: 10.1126/sciadv.abd1160, PMID: 33208372 PMC7673811

[ref90] Xu Z. Niu L. Li L. Taylor J. A. (2016). ENmix: a novel background correction method for Illumina HumanMethylation450 BeadChip. Nucleic Acids Res. 44:e20. doi: 10.1093/nar/gkv907, PMID: 26384415 PMC4756845

[ref91] Yang H. M. Yang S. Huang S. S. Tang B. S. Guo J. F. (2017). Microglial activation in the pathogenesis of Huntington's disease. Front. Aging Neurosci. 9:193. doi: 10.3389/fnagi.2017.00193, PMID: 28674491 PMC5474461

[ref92] Zhang Z. Wiencke J. K. Kelsey K. T. Koestler D. C. Christensen B. C. Salas L. A. (2022). HiTIMED: hierarchical tumor immune microenvironment epigenetic deconvolution for accurate cell type resolution in the tumor microenvironment using tumor-type-specific DNA methylation data. J. Transl. Med. 20:516. doi: 10.1186/s12967-022-03736-6, PMID: 36348337 PMC9644569

[ref93] Zhou W. Laird P. W. Shen H. (2017). Comprehensive characterization, annotation and innovative use of Infinium DNA methylation BeadChip probes. Nucleic Acids Res. 45:e22. doi: 10.1093/nar/gkw967, PMID: 27924034 PMC5389466

[ref94] Zhou W. Triche T. J. Jr. Laird P. W. Shen H. (2018). SeSAMe: reducing artifactual detection of DNA methylation by Infinium BeadChips in genomic deletions. Nucleic Acids Res. 46:e123. doi: 10.1093/nar/gky691, PMID: 30085201 PMC6237738

[ref95] Zhu T. Liu J. Beck S. Pan S. Capper D. Lechner M.. (2022). A pan-tissue DNA methylation atlas enables in silico decomposition of human tissue methylomes at cell-type resolution. Nat. Methods 19, 296–306. doi: 10.1038/s41592-022-01412-7, PMID: 35277705 PMC8916958

