# Supplementary material for: Hierarchical deconvolution for extensive cell type resolution in the human brain using DNA methylation
Source: Front Neurosci. 2023 Jun 19;17:1198243. doi: 10.3389/fnins.2023.1198243 (PMC10315586; doi:10.3389/fnins.2023.1198243)
Supplement: Supplementary file 1 [file Data_Sheet_1.docx]

Supplementary Materials

**Hierarchical deconvolution for extensive cell type resolution in the human brain using DNA methylation**

Table of Contents

**Supplementary Figure 1.** Dispersion separability criterion between brain cell types across the tested libraries 2

**Supplementary Figure 2.** Absolute difference values between predicted and true cell proportions in the discovery data set across the tested libraries3

**Supplementary Figure 3.** Heatmaps of the methylation status of the HiBED CpGs across the layers in the deconvolution hierarchy4

**Supplementary Figure 4.** The number of overlapping CpGs across the four libraries in HiBED5

**Supplementary Figure 5.** Methylation status of selected CpG in the HiBED libraries for each cell type with associated functional gene6

**Supplementary Figure 6**. Performance comparisons across HiBED, CETS, and EpiSCORE of neuronal cell prediction on in-silico mixtures and FANS-measured bulk brain samples7

**Supplementary Figure 7.** HiBED performance on independent data of microglia from patients with various age groups and adult vein endothelial cells8

**Supplementary Figure 8.** HiBED and EpiSCORE performance comparisons on independent microglial samples from patients with psychiatric disorders 9

**Supplementary Figure 9.** Stacked bar plots of HiBED deconvolved cell proportions in four brain regions 10

**Supplementary Figure 10.** Aging effect on HiBED-predicted GABA proportion in cortical subregions in males11

**Supplementary Figure 11.** Aging effect on HiBED-predicted neuron proportion, GABA proportion, GLU proportion, and GABA to GLU ratio in cerebellum stratified by sex12

**Supplementary Figure 12.** HiBED-predicted glia-to-neuron ratio in four brain regions13

**Supplementary Figure 13.** HiBED-predicted glial cell composition in the human cortex14

**Supplementary Table 1.** Multivariable linear regression model results for investigating HiBED-predicted cell alteration in health conditions15

**
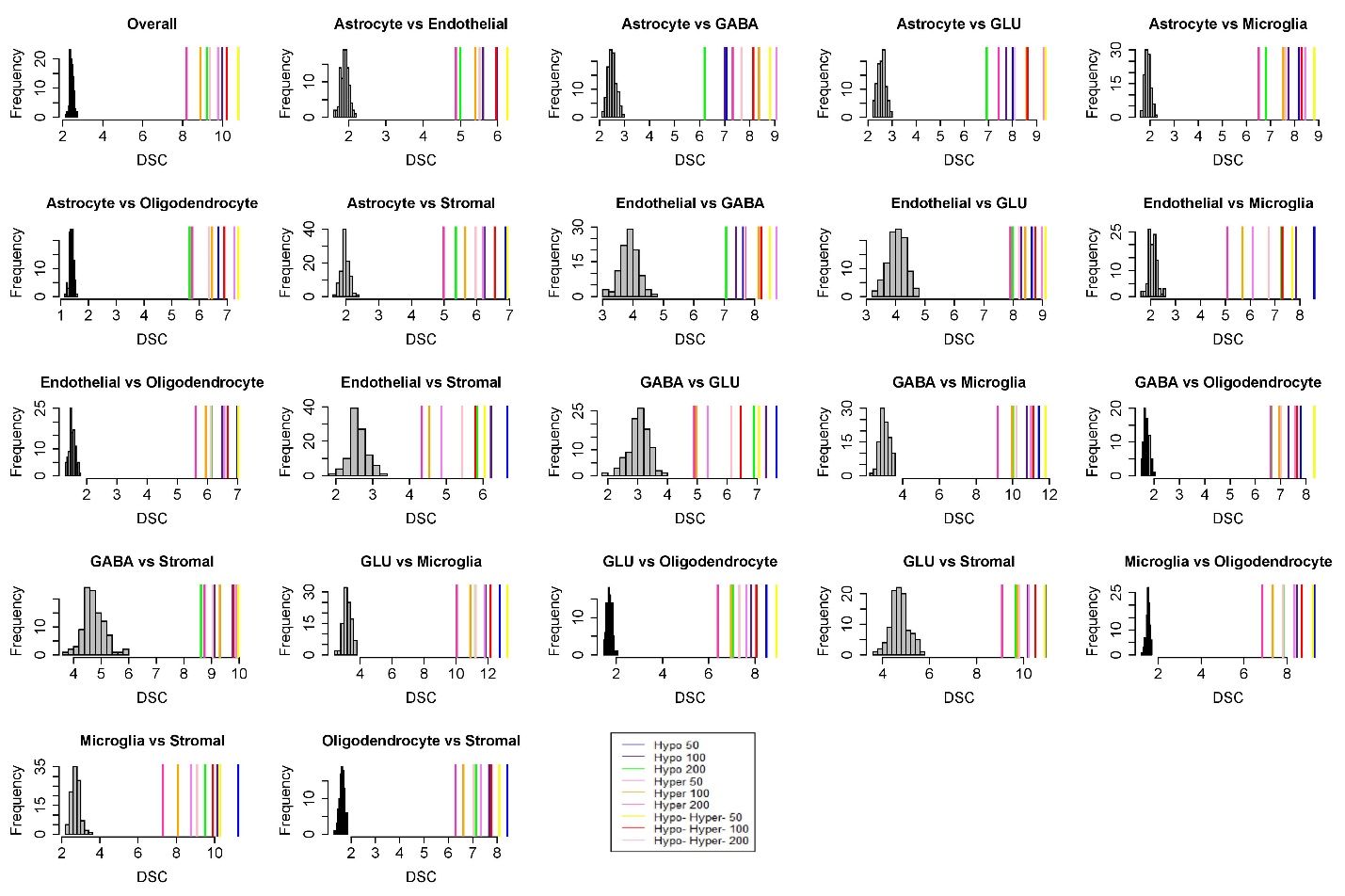
**

**Supplementary Figure 1.** Dispersion separability criterion between brain cell types across the tested libraries.

**
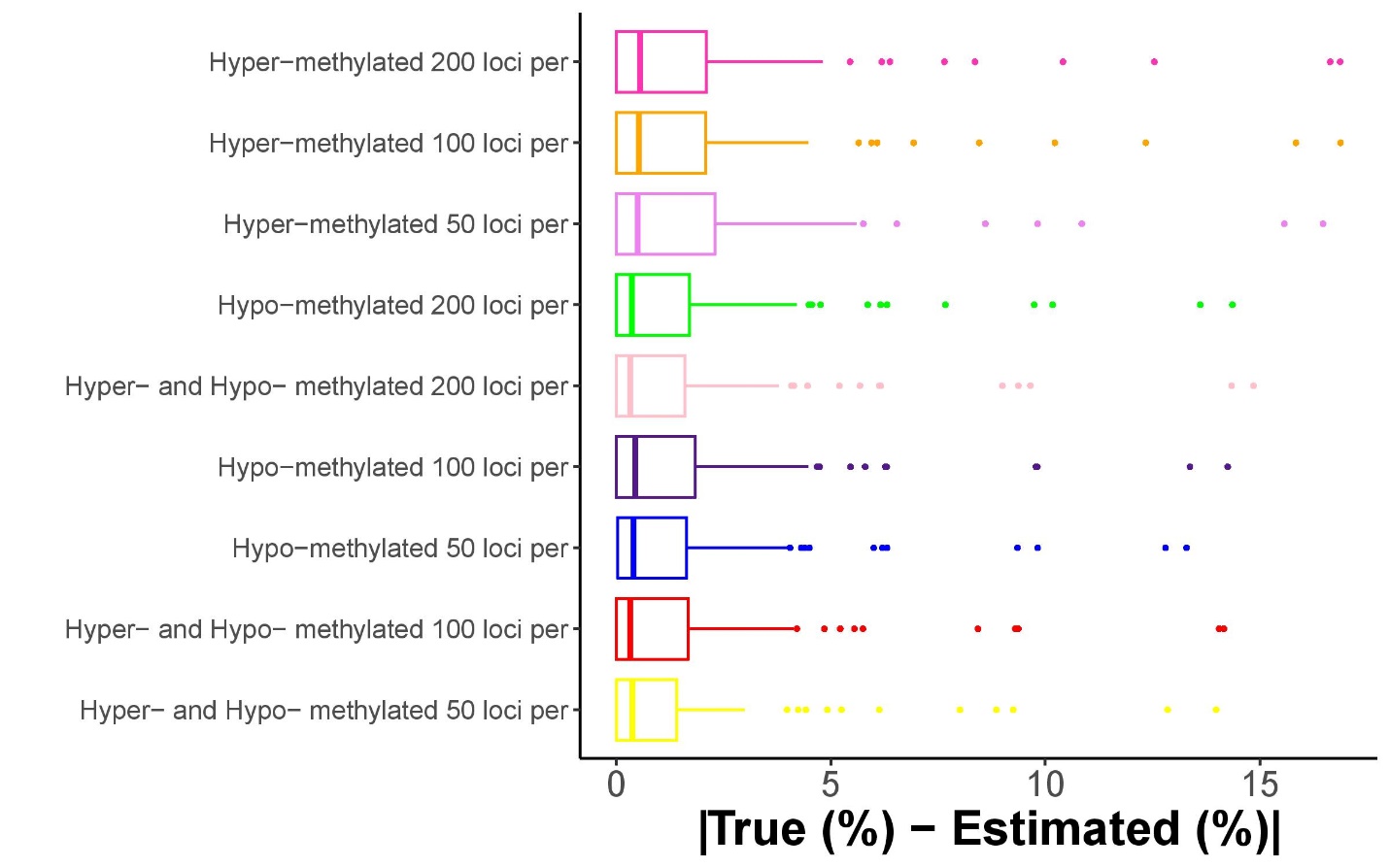
**

**Supplementary Figure 2.** Absolute difference values between predicted and true cell proportions in the discovery data sets across the tested libraries.


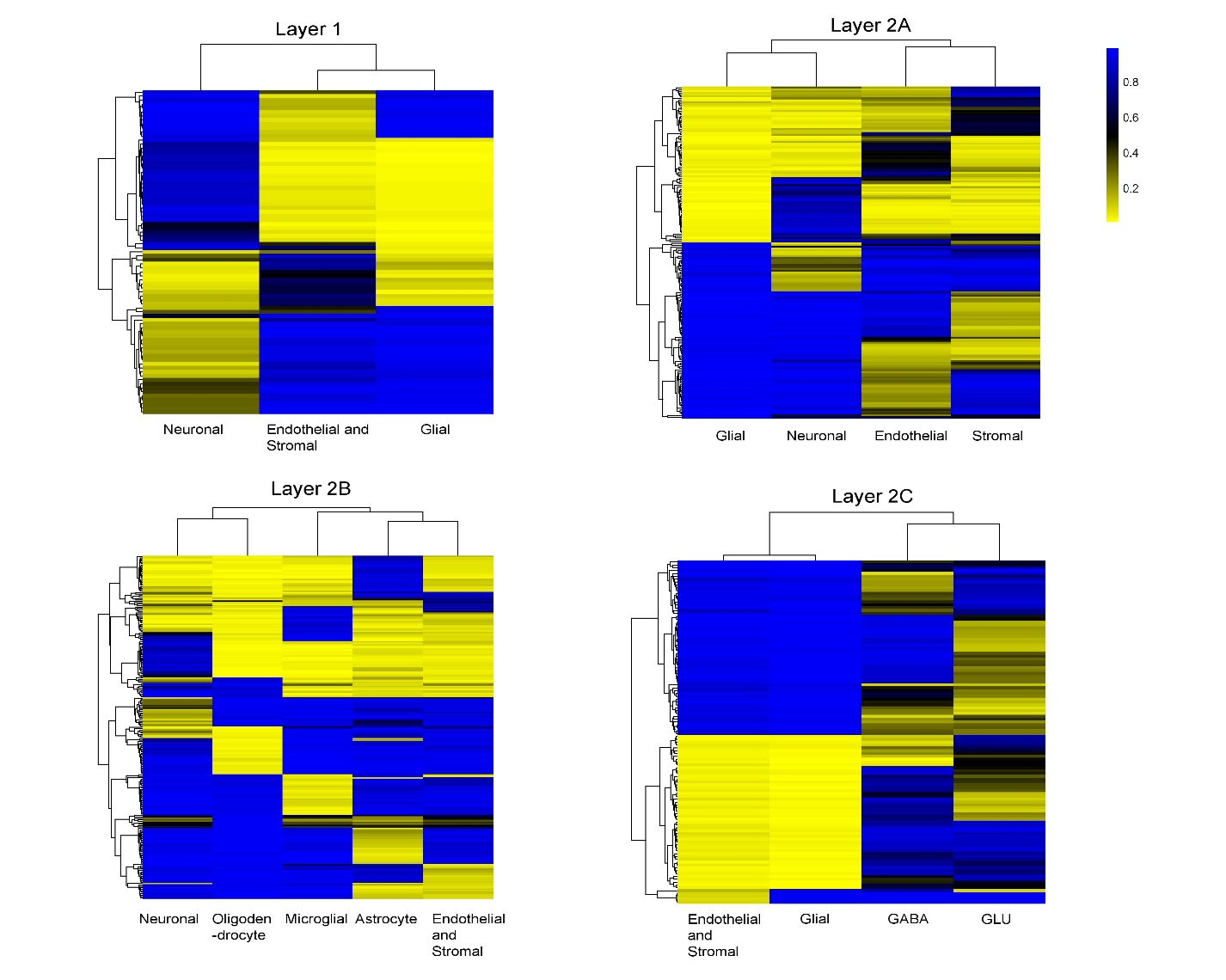
**Supplementary Figure 3**. Heatmaps of the methylation status of the HiBED CpGs across the layers in the deconvolution hierarchy.

**
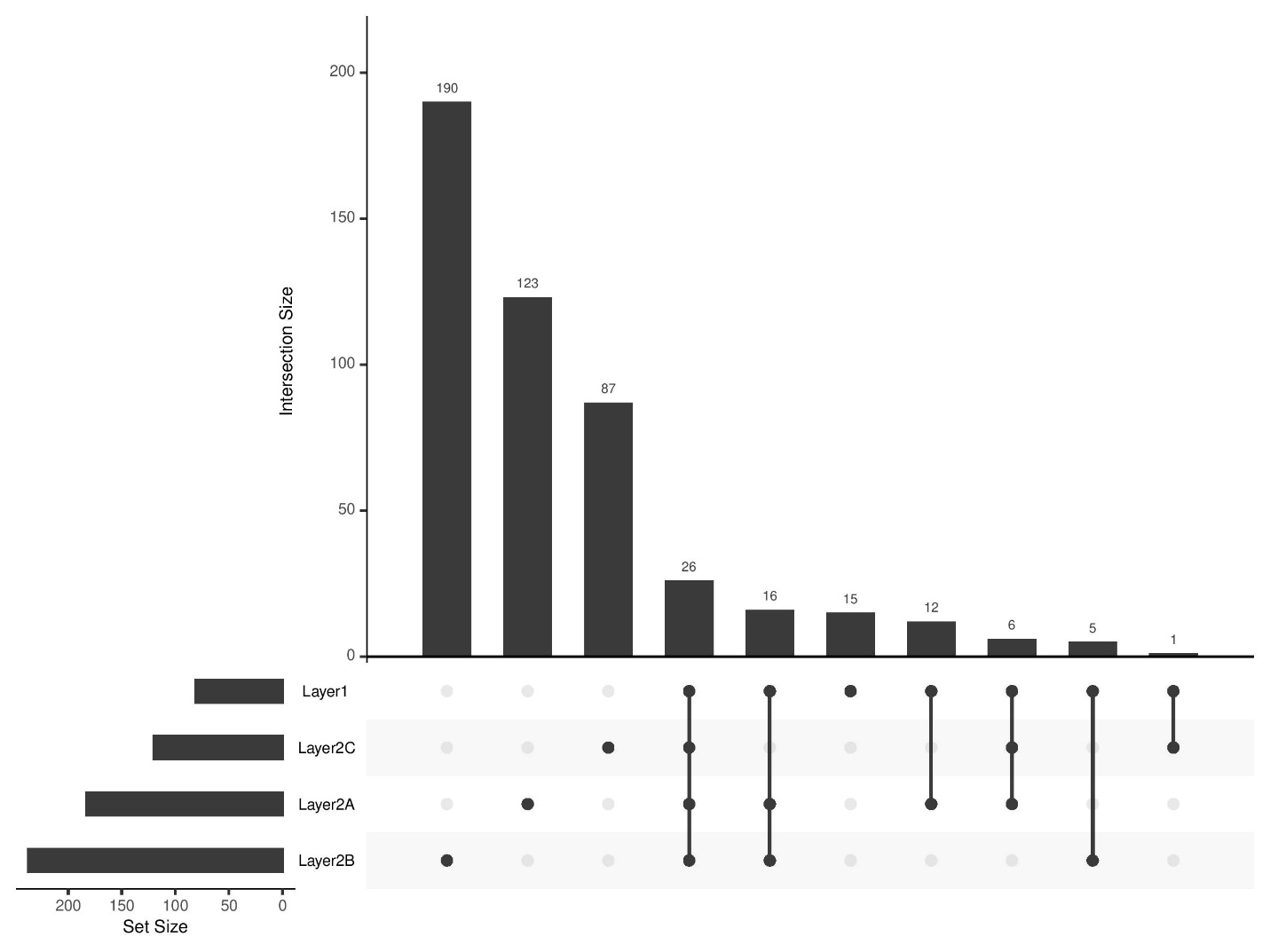
**

**Supplementary Figure 4.** The number of overlapping CpGs across the four libraries in HiBED.

**
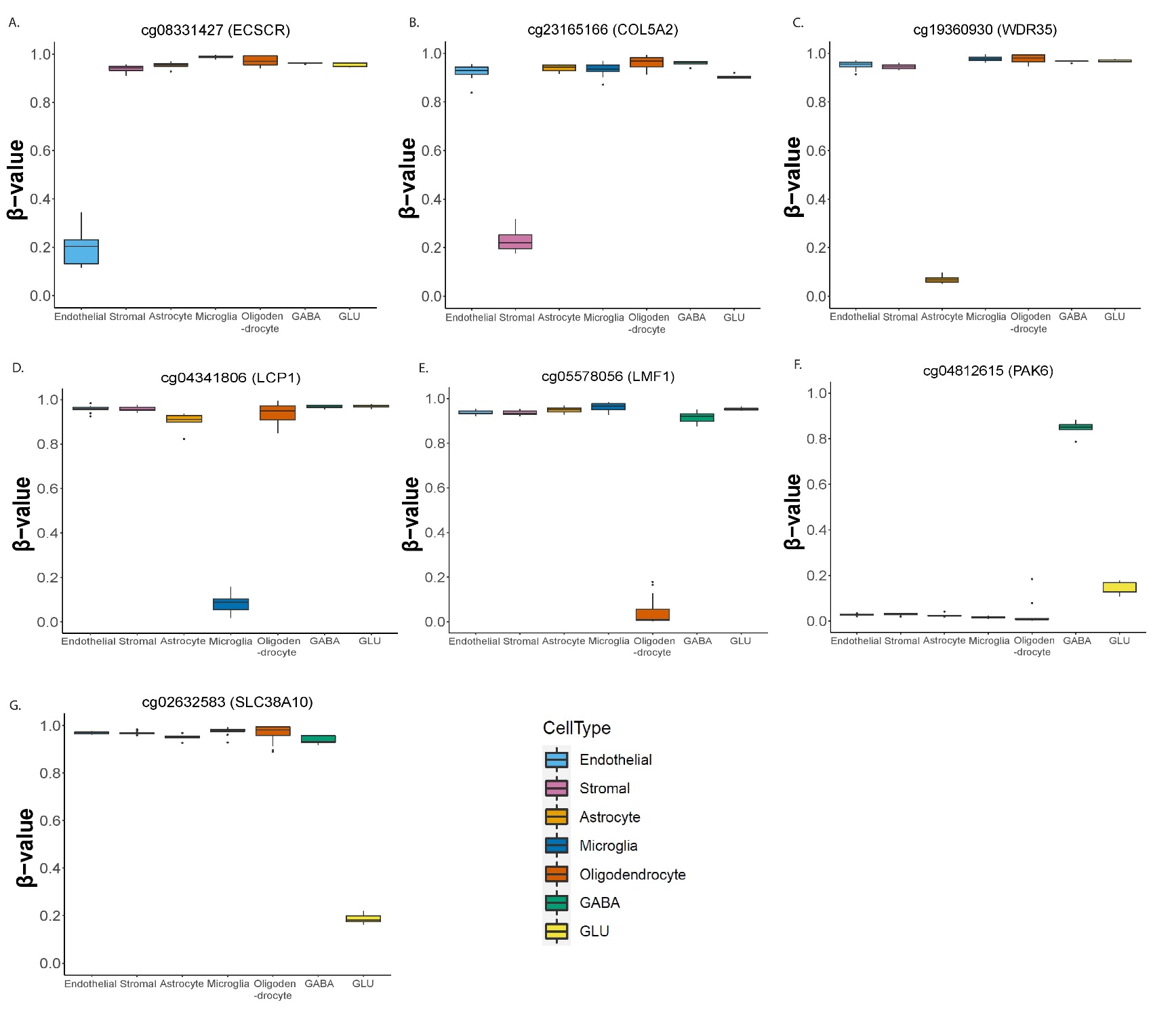
**

**Supplementary Figure 5.** Methylation status of selected CpG in the HiBED libraries for each cell type with associated functional gene.

**
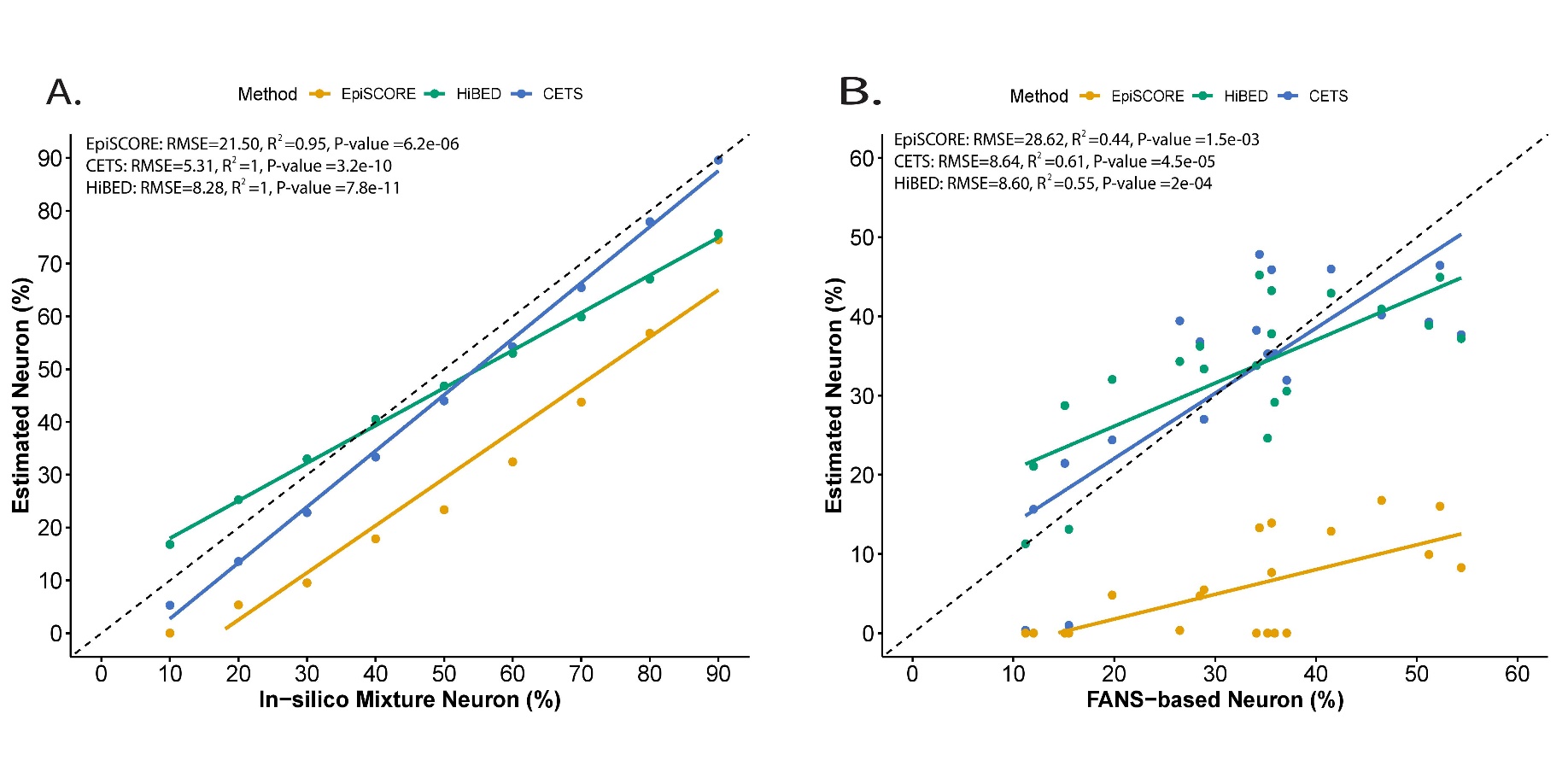
Supplementary Figure 6**. Performance comparisons across HiBED, CETS, and EpiSCORE of neuronal cell prediction on in-silico mixtures and FANS-measured bulk brain samples

**
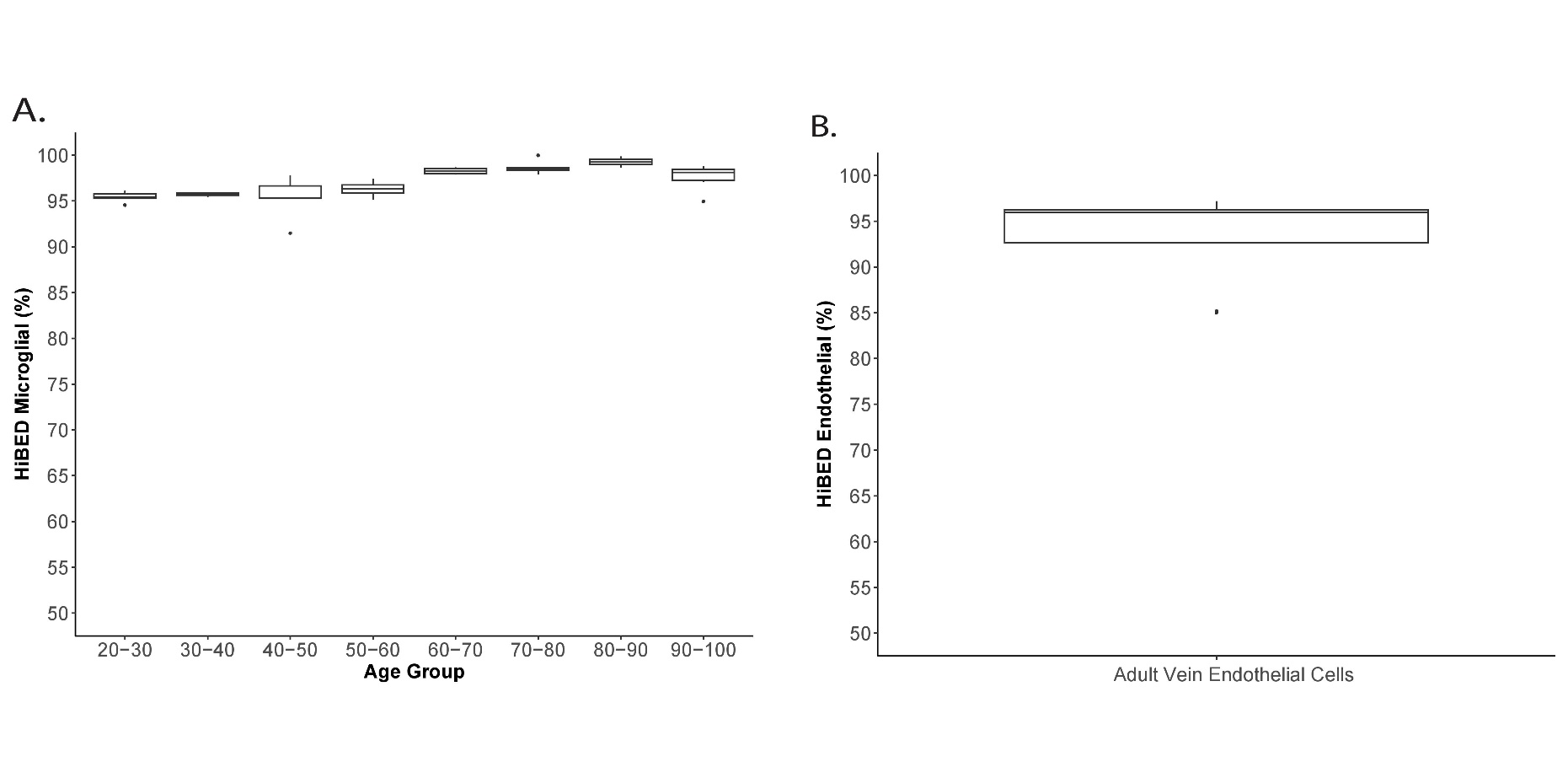
Supplementary Figure 7.** HiBED performance on independent data of microglia from patients with various age groups and adult vein endothelial cells

**
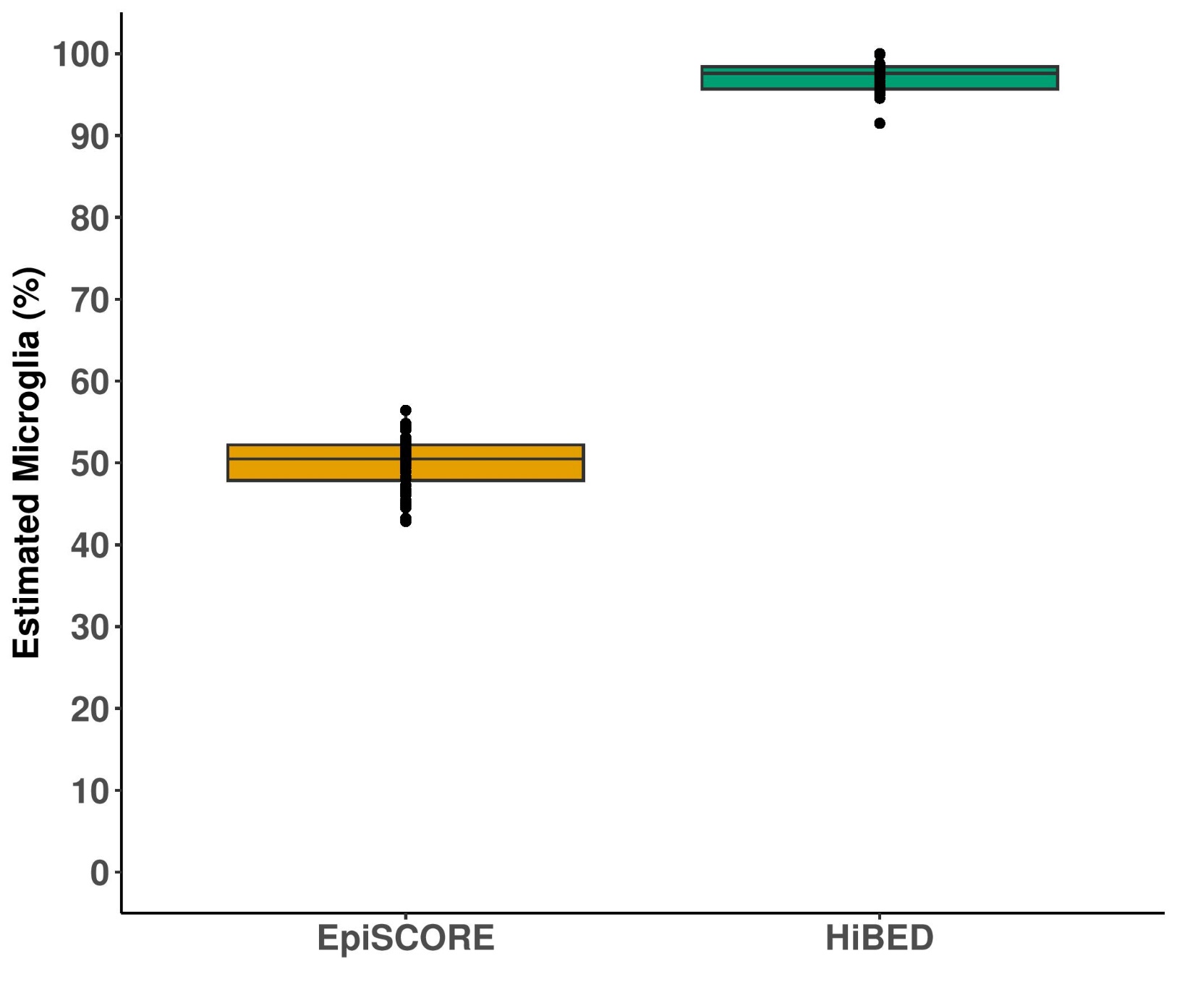
**

**Supplementary Figure 8.** HiBED and EpiSCORE performance comparisons on independent microglial samples from patients with psychiatric disorders.


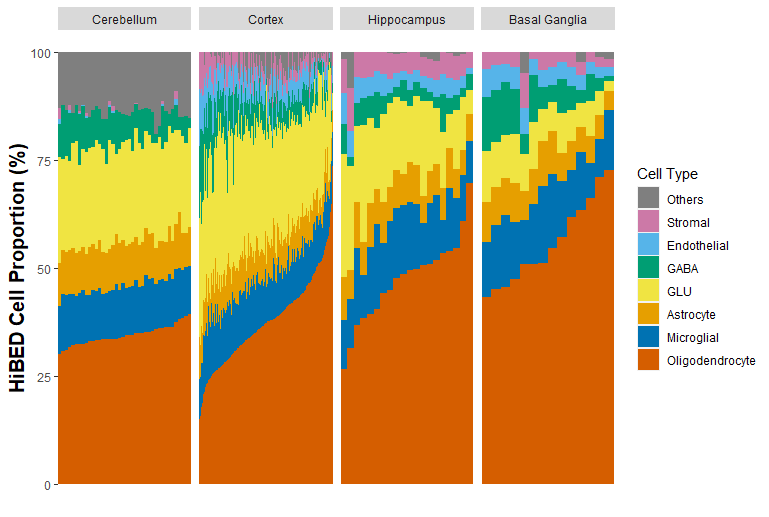
 **Supplementary Figure 9.** Stacked bar plots of HiBED deconvolved cell proportions in four brain regions.


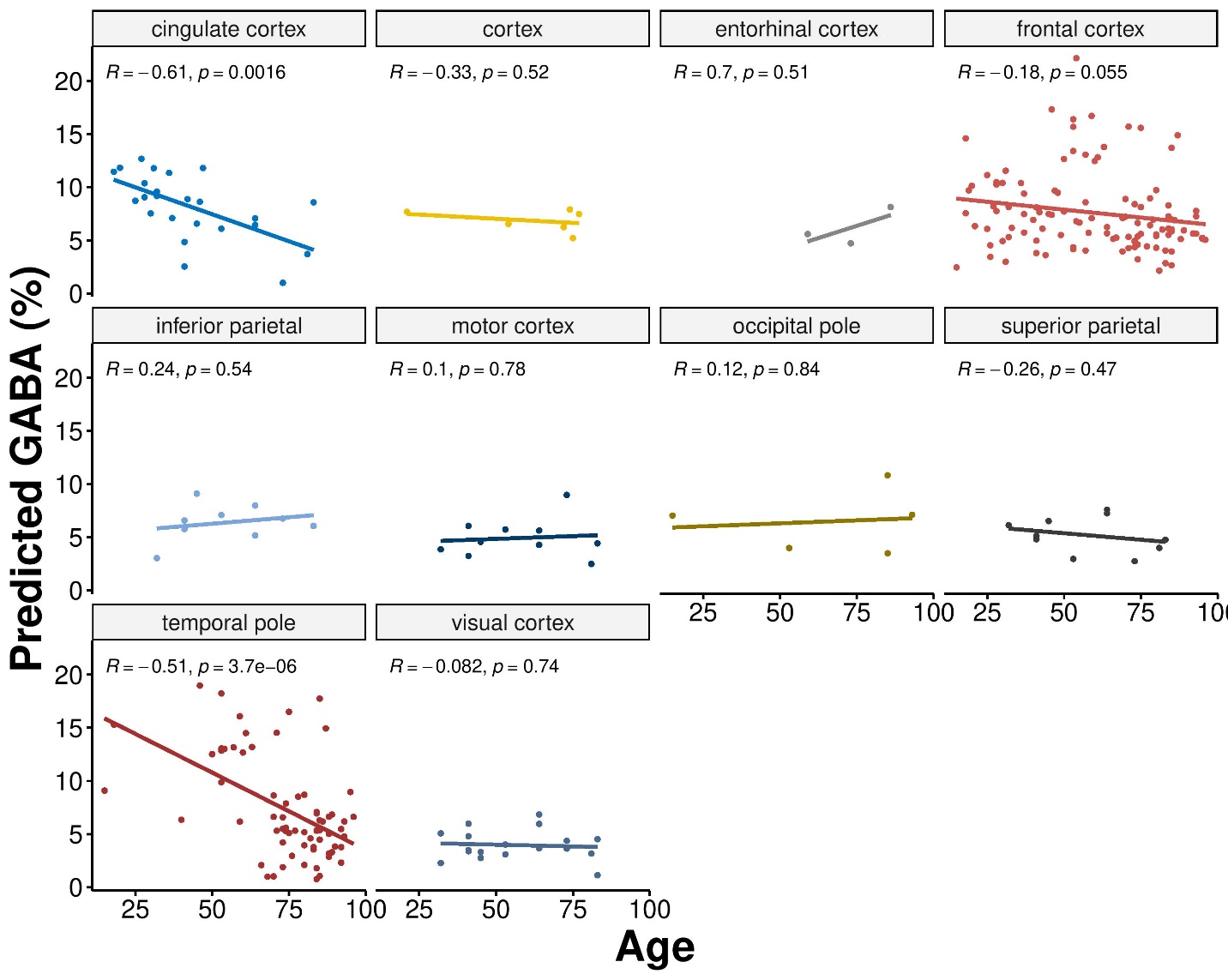


**Supplementary Figure 10.** Aging effect on HiBED-predicted GABA proportion in cortical subregions in males.

**
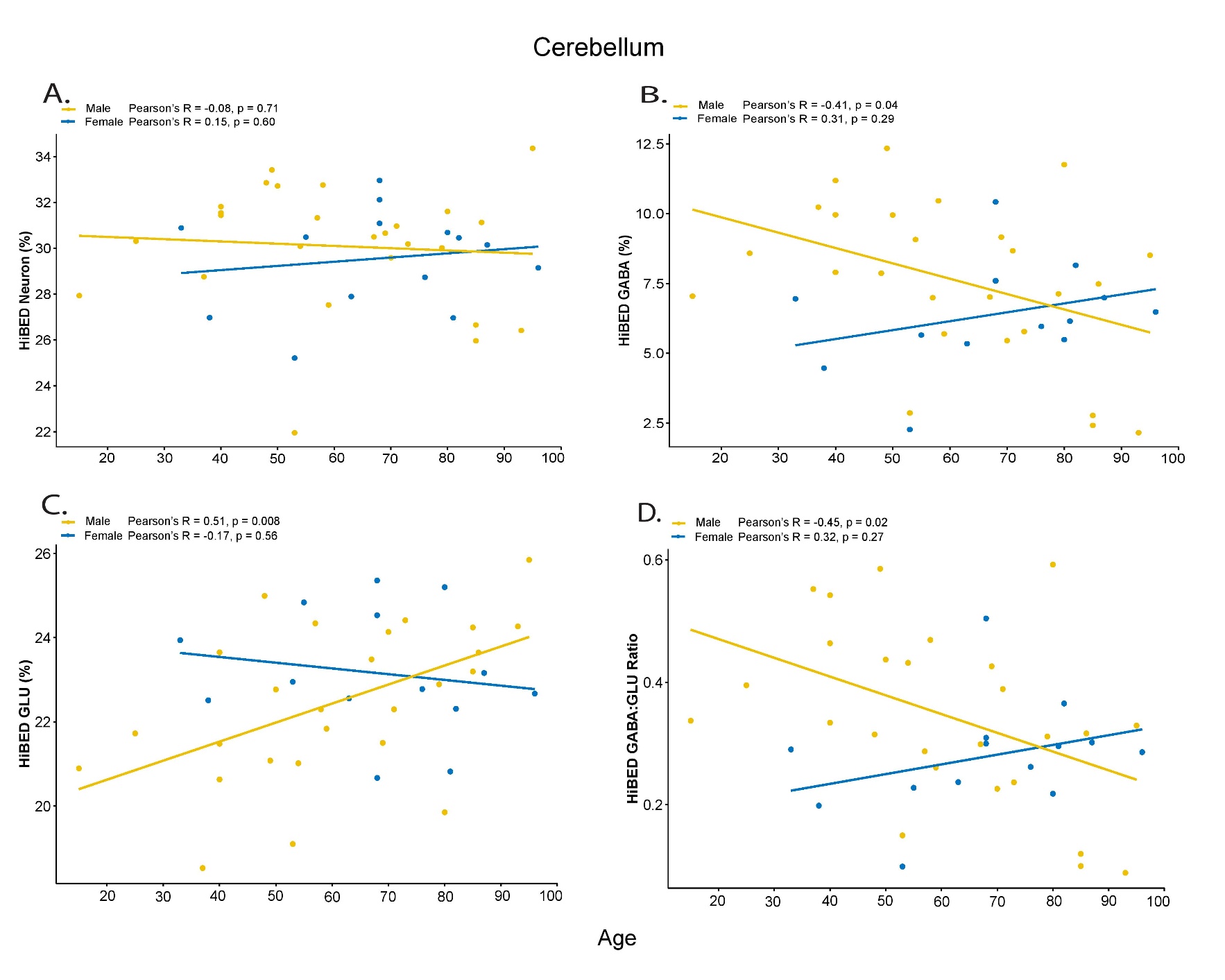
Supplementary Figure 11.** Aging effect on HiBED-predicted neuron proportion, GABA proportion, GLU proportion, and GABA to GLU ratio in cerebellum stratified by sex.


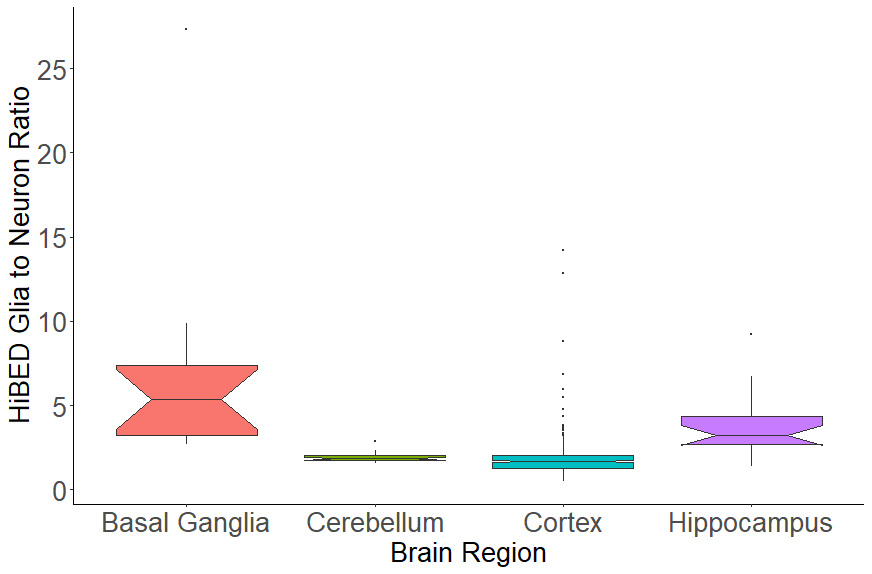
 **Supplementary Figure 12.** HiBED-predicted glia-to-neuron ratio in four brain regions.


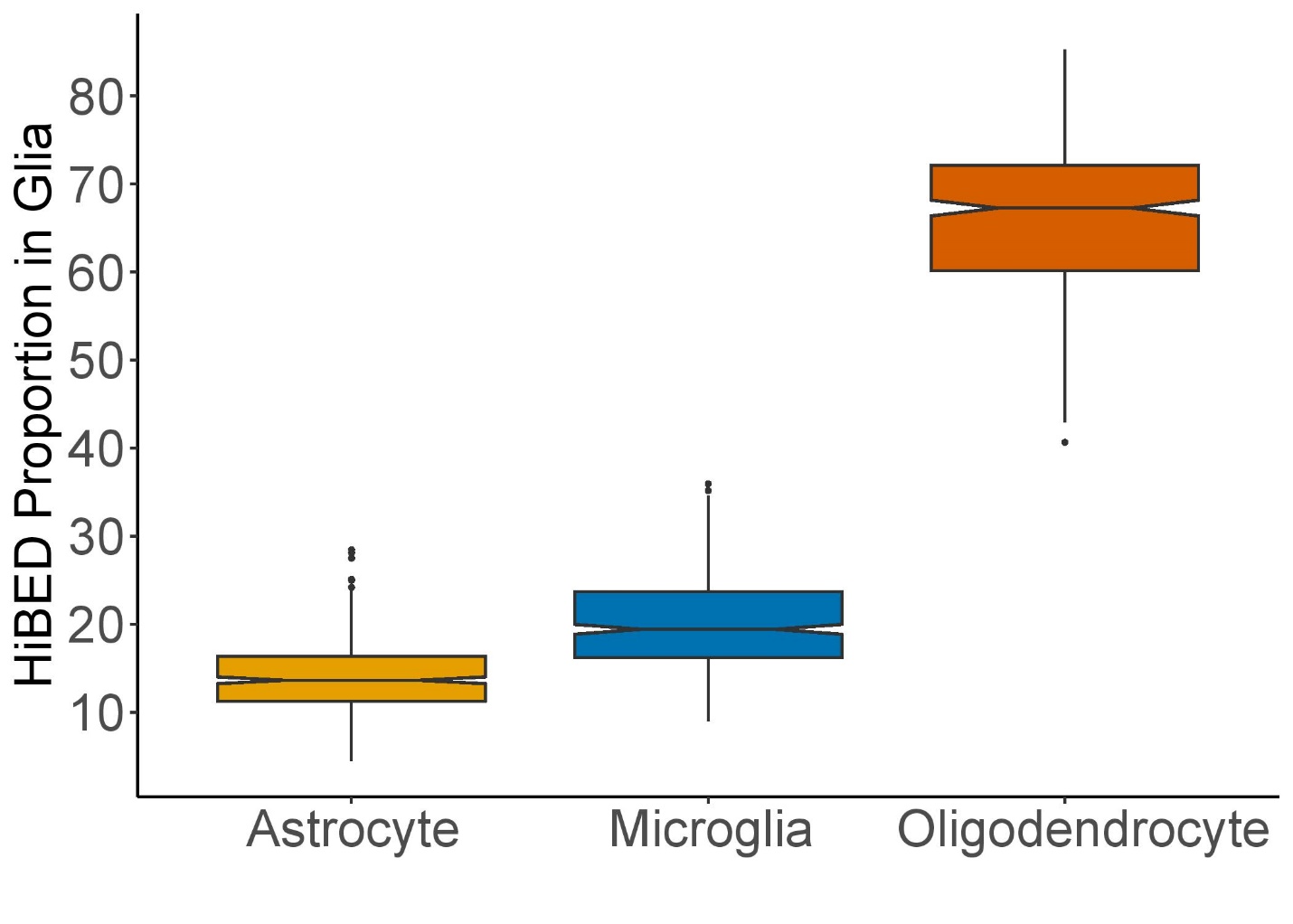


**Supplementary Figure 13.** HiBED-predicted glial cell composition in the human cortex.

**Supplementary Table 1.** Multivariable linear regression model results for investigating HiBED-predicted cell alteration in health conditions. **Δ** indicates the change of the cell proportion in cases compared to controls.
